# Recent advances in gene therapy for neurodevelopmental disorders with epilepsy

**DOI:** 10.1111/jnc.15168

**Published:** 2020-09-28

**Authors:** Thomas J. Turner, Clara Zourray, Stephanie Schorge, Gabriele Lignani

**Affiliations:** ^1^ Department of Clinical and Experimental Epilepsy UCL Queen Square Institute of Neurology London UK; ^2^ Department of Pharmacology UCL School of Pharmacy London UK

**Keywords:** disease models, epilepsy, gene therapy, ion channels, neurodevelopmental, synaptic proteins

## Abstract

Neurodevelopmental disorders can be caused by mutations in neuronal genes fundamental to brain development. These disorders have severe symptoms ranging from intellectually disability, social and cognitive impairments, and a subset are strongly linked with epilepsy. In this review, we focus on those neurodevelopmental disorders that are frequently characterized by the presence of epilepsy (NDD + E). We loosely group the genes linked to NDD + E with different neuronal functions: transcriptional regulation, intrinsic excitability and synaptic transmission. All these genes have in common a pivotal role in defining the brain architecture and function during early development, and when their function is altered, symptoms can present in the first stages of human life. The relationship with epilepsy is complex. In some NDD + E, epilepsy is a comorbidity and in others seizures appear to be the main cause of the pathology, suggesting that either structural changes (NDD) or neuronal communication (E) can lead to these disorders. Furthermore, grouping the genes that cause NDD + E, we review the uses and limitations of current models of the different disorders, and how different gene therapy strategies are being developed to treat them. We highlight where gene replacement may not be a treatment option, and where innovative therapeutic tools, such as CRISPR‐based gene editing, and new avenues of delivery are required. In general this group of genetically defined disorders, supported increasing knowledge of the mechanisms leading to neurological dysfunction serve as an excellent collection for illustrating the translational potential of gene therapy, including newly emerging tools.

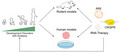

## Graphical abstract

Neurodevelopmental disorders with epilepsy (NDD + E) are caused by mutations in neuronal genes fundamental to brain development. These disorders have severe symptoms ranging from intellectually disability, social impairments and seizures. In this review we group the genes linked to NDD + E with different neuronal functions: transcriptional regulation, intrinsic excitability and synaptic transmission. We review the uses and limitations of current models, and how different gene therapy strategies are being developed to treat NDD + E. We highlight where gene replacement may not be a treatment option, and where innovative therapeutic tools, such as CRISPR‐based gene editing, and new avenues of delivery are required.

**Abbreviations**
ADNFLE: autosomal‐dominant nocturnal frontal lobe epilepsy
AED: anti‐epileptic drug
AIS: axon initial segment
AMPAR: AMPA receptor
AP: action potential
AS: angelman syndrome
ASD: autism spectrum disorder
BFN/IS: benign familial neonatal/infantile seizures
BK channels: calcium‐activated potassium channels
CDD: CDKL5‐deficiency disorder
CNS: central nervous system
DS: Dravet syndrome
E: epilepsy
EAS: epilepsy‐aphasia spectrum
EE: epileptic encephalopathy
EIMFS: epilepsy of infancy with migrating focal seizures
FS+: febrile seizure plus
FXS: fragile X syndrome
GEFS+: generalized epilepsy with febrile seizure plus
GoF: gain‐of‐function
hCS: human cortical spheroids
hESC: human embryonic stem cell
hiPSC: human induced pluripotent stem cell
hSS: human subpallial spheroids
ID: intellectual disability
KO: knockout
LoF: loss‐of‐function
NDD + E: neurodevelopmental disorders with epilepsy
NDD: neurodevelopmental disorders
NMDAR: NMDA receptor
PKD: paroxysmal kinesigenic dyskinesia
PKD/IC: paroxysmal kinesigenic dyskinesia with infantile convulsions
PTV: protein truncating variant
PTZ: pentylenetetrazole
PV+: parvalbumin positive
RTT: Rett syndrome
SCN: voltage‐gated sodium channel
SEN: subependymal nodule
SST+: somatostatin positive
SV: synaptic vesicle
TSC: tuberous sclerosis complex

## 1 NEURODEVELOPMENTAL DISORDERS WITH EPILEPSY AND EPILEPTIC ENCEPHALOPATHIES

Neurodevelopmental disorders (NDDs) are a broad and diverse group of behavioural disorders, including autism spectrum disorders (ASDs) and intellectual disability (ID), that are defined by significant impairment in one or more domains of functioning for example social interactions, cognition, language and/or motor skills (Ismail & Shapiro, 2019). NDDs are commonly associated with severe and intractable epilepsy, with approximately 26% of patients reporting seizures as a comorbidity (Association, 2013).

In contrast, epileptic encephalopathies (EEs) are a broad group of syndromes characterized by early onset epilepsies that are often comorbid with NDDs (McTague, Howell, Cross, Kurian, & Scheffer, 2016). The term ‘epileptic encephalopathy’ denotes a process by which epileptic activity adversely affects brain function over and above the underlying aetiology, such that seizures can be the direct cause of developmental delay and cognitive impairment. However, evidence of such a process occurring in many of the syndromes currently defined as EEs is still lacking and eliminating epileptic activity through the use of anti‐epileptic drugs (AEDs) is often not sufficient to prevent developmental delay and cognitive impairments (Howell, Harvey, & Archer, 2016). This highlights the importance of developing novel therapeutics that target the specific genetic cause of the disease. By restoring correct protein function early on, gene therapies provide the possibility of preventing both developmental delay and epileptic activity (Wykes & Lignani, 2018).

In this review, we will use the general term neurodevelopmental disorders with epilepsy (NDD + E), which includes any disorder affecting the development of the brain associated with some level of epileptic activity, regardless of whether epilepsy is seen as a primary pathological process or a comorbidity. Thus, NDD + E comprise a heterogeneous group of disorders that are frequently caused by de novo mutations in single genes.

**Figure 1:**
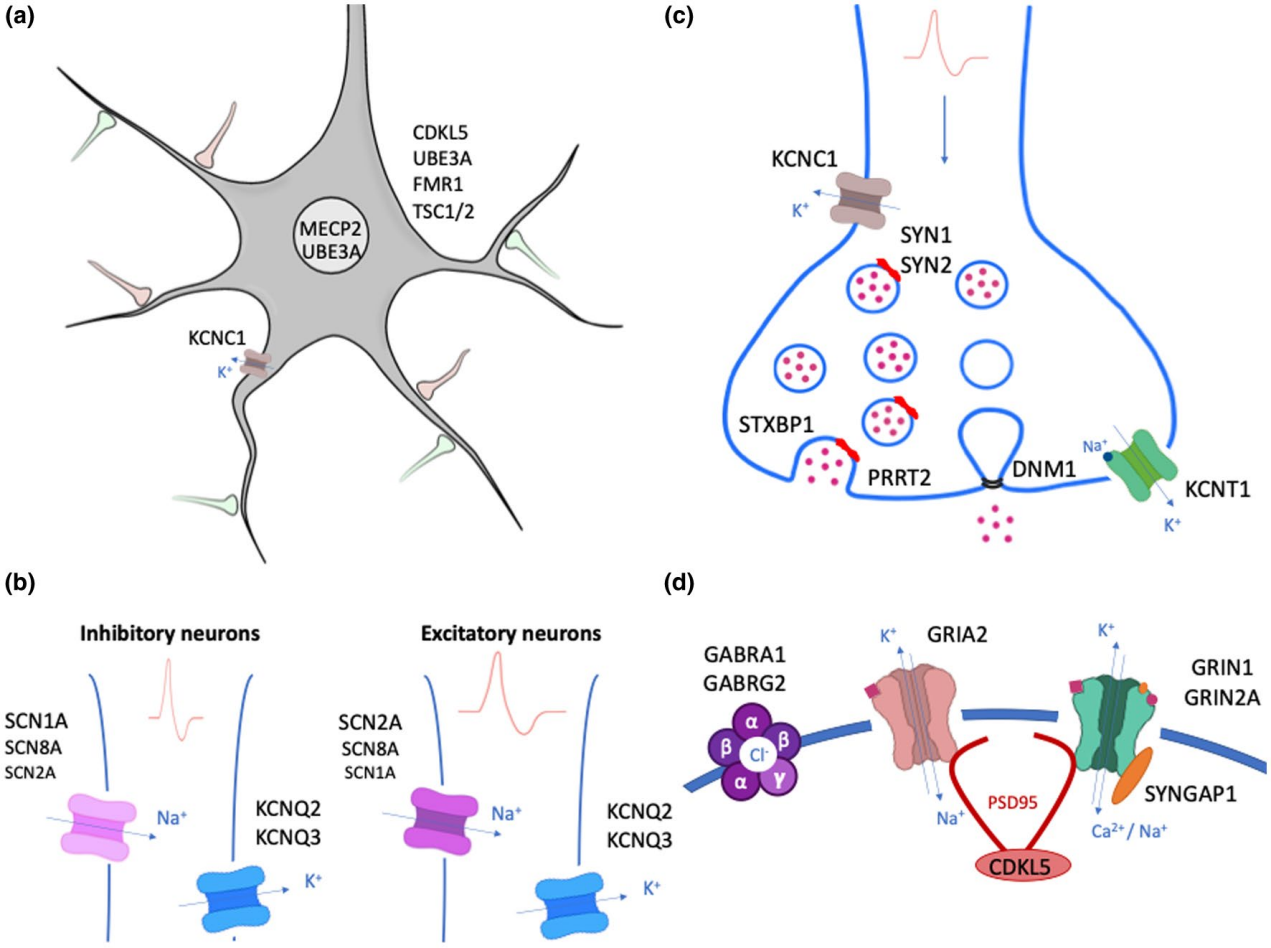
Mutated genes in NDD + E. Gene names are drawn where their encoded proteins function in the cell body (A), axon initial segment (B), pre‐synaptic (C) and post‐synaptic (D) terminals. In (A), genes outside the neuron represent ubiquitous expression. In (B), gradient expression of SCN channels is indicated by different font sizes

Recent advances in next‐generation sequencing have allowed the identification of more than 100 genes associated with NDD + E, which encode proteins with diverse cellular functions (Heyne et al., 2019; Symonds & McTague, 2020). In this review, we do not aim to describe all the genes associated with NDD + E, but rather to explore how three different categories of genes linked to these disorders are modelled, and how the different models have advantages and disadvantages for each category of genes. Broadly speaking we consider mutations that affect genes that alter neuronal activity indirectly (i.e. through altering the regulation of other genes), directly change neuronal excitability by targeting ion channels and alter synaptic properties (Figure 1).

### 1.1 Mutations that cause NDD + E by changing gene expression levels

During development, gene expression has to be tightly regulated to achieve the orderly sequence of events resulting in the correct formation and maturation the nervous system (Kang et al., 2011). Mutations in genes affecting transcriptional, translational or post‐translational mechanisms can have important pathological effects on brain development and function, even where these genes do not directly change neuronal activity. Three examples are Rett syndrome (RTT), Fragile X syndrome (FXS) and Angelman syndrome (AS), all of which appear to change overall neuronal function indirectly by modifying the expression of other genes during development.

RTT, the primary cause of ID and ASD in girls worldwide, was found to be caused by loss‐of‐function (LoF) mutations in the X‐linked gene *MECP2* more than 20 years ago (Amir et al., 1999; Singer & Naidu, 2001). *MECP2* encodes the methyl‐CpG binding protein 2 (MeCP2), a member of the methyl‐CPG‐binding domain (MBD) protein family that binds methylated cytosines. MeCP2 is most abundant in the brain where it was found interacting with methylated sites to repress the transcription of long genes (Gabel et al., 2015). Further studies found that MeCP2 differentially regulates gene expression in inhibitory and excitatory neurons, revealing a contextual role for MeCP2 in mediating RTT pathology (Johnson et al., 2017). MeCP2 is now considered a global regulator of chromatin structure that is required to fine‐tune the gene expression (Skene et al., 2010). The loss of MeCP2 in RTT leads to subtle but widespread changes in expression during late‐embryonic and post‐natal development that result in aberrant neuronal maturation and synapse formation. This leads to the onset of RTT symptoms after the first year of life, characterized by developmental arrest or regression, with a deterioration in communication, social and fine motor skills (Singer & Naidu, 2001).

FXS, the most common monogenic cause of ASD and ID (Richter, Bassell, & Klann, 2015), is caused by expansion of trinucleotide cytosine‐cytosine‐guanine (CGG) repeats in the *FMR1* gene leading to transcriptional silencing of the *FMR1* locus and loss of FMRP protein function. Roughly half of all FXS patients show paroxysmal abnormalities on EEG, with 20% of FXS patients developing a form of epilepsy. Approximately 25% of patients are refractory to anti‐epileptic drugs and will continue to have seizures into adulthood (Hagerman & Stafstrom, 2009). FMRP is an important regulator of protein translation and its disruption leads to the dysregulation of hundreds of proteins affecting synaptic plasticity and connectivity in the developing brain (Richter et al., 2015). Analysis of the FMRP transcriptome has uncovered hundreds of potential mRNA targets that encode pre‐ and post‐synaptic proteins as well as a number of ion channels. Notably, FMRP has been shown to directly interact with Kv3.1, Kv4.2, Cav2.2 and BK channels, altering the channel properties and regulating their membrane trafficking. Those interactions can directly alter neuronal excitability, possibly contributing to the patients’ phenotype (Ferron, 2016).

AS is another severe NDD + E, which is characterized by microcephaly, seizures, ataxia, muscular hypotonia with hyperreflexia and motor delay (Buiting, Williams, & Horsthemke, 2016). LoF mutations in *UBE3A*, an E3 ubiquitin ligase that conjugates ubiquitin groups to proteins in order to target them for degradation, were found to cause AS (Sell & Margolis, 2015). The mechanisms by which *UBE3A* LoF can lead to cortical hyperexcitability and epilepsy had long remained elusive, until recently one mechanism was suggested by the discovery that a lack of UBE3A‐mediated degradation of large conductance calcium‐activated potassium channels (BK) channels and resulting augmented BK channel activity leads to increased intrinsic cellular excitability (Sun et al., 2019).

Tuberous sclerosis (TSC) is a multisystem disorder caused by mutations in *TSC1* or *TSC2*, genes encoding for hamartin and tuberin respectively (Curatolo, Bombardieri, & Jozwiak, 2008). Tuberin and hamartin are both critical regulators of the mTOR pathway and heterozygous LoF mutations are sufficient to dysregulate cell proliferation and differentiation resulting in CNS lesions such as subependymal nodules (SENs) and cortical tubers (Curatolo et al., 2008). Double‐hit somatic mutations causing homozygosity through copy‐neutral loss‐of‐heterozygosity can also occur and are found in some but not all lesions (Martin et al., 2017). Around 85% of patients with TSC develop NDD + E phenotypes including cognitive impairment, behavioural problems, autism and epilepsy. Epilepsy usually begins in the first year of life and is thought to mainly arise from cortical tubers (Curatolo et al., 2018).

### 1.2 Disorders associated with direct changes in neuronal excitability: mutations in ion channels

Ion channels are transmembrane proteins that regulate ion flux across cell membranes and play a key role in controlling the electrical properties and excitability of neurons (Hille, 2001). In the 1990s, the discovery of the first disease‐causing monogenetic mutations in ion channels led to the identification of a new group of diseases, known as channelopathies. Genetic channelopathies are a heterogeneous group of diseases as their clinical features and age of presentation are dependent not only on the physiological role of the ion channel in question, but also on the specific spatial and temporal expression patterns of the gene (Kullmann, 2010).

#### 1.2.1 Voltage‐gated sodium channels (SCN1A, SCN2A, SCN8A)

Genetic variants in *SCN1A*, *SCN2A* and *SCN8A* encoding the α subunits of brain‐expressed voltage‐gated sodium channels (SCNs) are some of the most frequent monogenic causes of NDD + E (Brunklaus & Lal, 2020). SCNs are transmembrane complexes that consist of one α subunit and one or more auxiliary β subunits (Catterall, 2000). As they allow for the rapid influx of Na^+^ ions upon membrane depolarization, SCNs are critical for the generation of action potentials (APs) at the axon initial segment (AIS) and their propagation along the axon.

Mutations in *SCN1A* resulting in Nav1.1 LoF are associated with a spectrum of phenotypic severity, from mild missense mutations causing febrile seizures plus (FS+) to more severe missense and protein truncating variants (PTVs) resulting in generalized epilepsy with febrile seizures plus (GEFS+) and Dravet syndrome (DS) (Catterall, Kalume, & Oakley, 2010). Nav1.1 is preferentially expressed at the AIS of GABAergic interneurons where it plays a critical role in AP generation (Yu et al., 2006). Nav1.1 LoF leads to reduced sodium current densities and decreased excitability of inhibitory neurons, thus tipping the excitation–inhibition balance towards hyperexcitability and causing severe, intractable epilepsy. As epilepsy is a result of sodium channel LoF, sodium channel‐blocking drugs such as carbamazepine and lamotrigine exacerbate the disorder and are contraindicated.

Both gain‐of‐function (GoF) and LoF variants in *SCN2A* have been associated with a spectrum of NDD + E, including benign familial neonatal‐infantile epilepsy (BFNIE), Ohtahara syndrome, Lennox‐Gastaut syndrome and West syndrome (Brunklaus et al., 2020; Wolff et al., 2017). LoF variants in *SCN2A* have also been identified in patients with later onset epilepsy and in patients with intellectual disability, ASD and schizophrenia without seizures (Carroll et al., 2016; Codina‐Solà et al., 2015; Wolff et al., 2017). It is possible that LoF mutations are relatively better tolerated than GoF as there may be more channels present than absolutely needed, or also that they may affect the neuronal excitability of different neurons at specific developmental stages, which could explain differences in seizure onset (Brunklaus et al., 2020; Wolff et al., 2017).

Similar to *SCN2A*, GoF variants in *SCN8A* have been reported in a spectrum of disorders ranging from benign infantile seizures to severe NDD + E (Larsen et al., 2015). LoF variants in *SCN8A* have also been identified in patients with intellectual disability and myoclonus without seizures (Wagnon et al., 2017; Wagnon et al., 2018). *SCN2A* and *SCN8A* encode Nav1.2 and Nav1.6, respectively, two sodium channel subunits that are predominantly expressed at the AIS and nodes of Ranvier of excitatory neurons and play a crucial role in AP propagation early in development. While they have similar function, *SCN2A* is expressed earlier in development than *SCN8A*, which explains the earlier age of onset in *SCN2A*‐related epilepsies (median = 13 days), compared to *SCN8A* patients (median = 4 months; Brunklaus et al., 2020).

#### 1.2.2 Voltage‐gated potassium channels (KCNQ2, KCNQ3, KCNT1, KCNC1)

Potassium channels are the largest ion channel group mostly expressed in the central nervous system (CNS). Voltage‐gated potassium channels are composed of four homologous pore‐forming subunits, each subunit containing six transmembrane α‐helices, of which the S4 segment acts as a voltage sensor (Kullmann, 2010).

*KCNQ2* and *KCNQ3* encode Kv7.2 and Kv7.3, respectively, two subunits that co‐assemble to form a slowly activated channel mediating the M current, which is particularly important during development for controlling network hyperexcitability (Wang et al., 1998). Kv7.2 and Kv7.3 are expressed at the AIS of both excitatory and inhibitory neurons. In excitatory neurons, loss of *KCNQ2* leads to a decreased medium afterhyperpolarization and long‐lasting depolarization resulting in overall increased excitability. Interestingly, deletion of *KCNQ2/3* in inhibitory neurons also leads to increased excitability through homeostatic potentiation of excitatory transmission (Soh et al., 2018). Therefore, LoF mutations in those channels would overall result in neuronal hyperexcitability and seizures. *KCNQ2* missense and truncating variants have been associated with the self‐limited BFNIE, as well as more severe NDD + E, such as Ohtahara syndrome, which do not currently have satisfactory treatment options (McTague et al., 2016; Shellhaas et al., 2017). Less commonly, *KCNQ3* variants have also been associated with BFNIE and recently a homozygous LoF variant was found in a patient with neonatal‐onset epilepsy and intellectual disability (Lauritano et al., 2019).

One of the most severe NDD + E, epilepsy of infancy with migrating focal seizures (EIMFS), is associated with de novo GoF mutations in *KCNT1*, encoding the sodium‐activated potassium channel subunit KCa4.1 (Barcia et al., 2012; McTague et al., 2018). Mutations in *KCNT1* have also been associated with a familial focal epilepsy syndrome, autosomal‐dominant nocturnal frontal lobe epilepsy (ADNFLE), as well as Ohtahara and West syndrome (Heron et al., 2012; Ohba et al., 2015). GoF *KCNT1* pathogenic variants are known to cause pleiotropic effects, although genotype–phenotype correlations are still unclear, with at least one mutation reportedly associated with both EIMFS and ADNFLE (Borlot et al., 2020).

Recent mutations in *KCNC1*, encoding the voltage‐gated potassium channel Kv3.1, have been associated with NDD + E. Kv3.1 is expressed in inhibitory neurons and it is fundamental for the fast‐spiking nature of these neurons. Although, dominant‐negative mutations in *KCNC1* leading to progressive myoclonus epilepsy have already been reported, new de novo recurrent mutations have been identified that cause NDD with and without an epileptic phenotype (Cameron et al., 2019; Park et al., 2019).

### 1.3 Mutations causing NDD + E that affect synaptic transmission

Synaptic transmission is a fundamental process for neuronal communication, network formation during development and plasticity associated with behaviour (Südhof, 2018). It is not surprising that mutations in proteins involved in this neuronal function lead to NDD + E.

Mutations have been found in proteins expressed at both pre‐ and post‐synaptic terminals. Synaptic proteins are highly complex collection of proteins that interact to allow correct function of the most abundant and defining feature of the human brain, synapses (Bayés et al., 2011). The human synapse proteome, or synaptome, is thought to comprise no less than 2000 proteins. Mutations in the genes encoding the synaptome have been found to cause 130 brain diseases, now regrouped under the collective term ‘synaptopathies’ (Grant, 2012). Synaptopathies can result in neurodevelopmental and psychiatric disorders such as ASD, ID or schizophrenia, but mutations in the synaptome have also been found to cause NDD + E.

#### 1.3.1 Pre‐synaptic terminal

*STXBP1* encodes Stxbp1/Munc18‐1, a protein involved in synaptic vesicle docking, priming and fusion through interactions with SNAREs (Rizo & Xu, 2015). De novo heterozygous mutations in *STXBP1* can cause some of the most severe forms of NDD + E, such as Ohtahara syndrome (Saitsu et al., 2010), West syndrome (Deprez et al., 2010), Lennox‐Gastaut syndrome (Allen et al., 2013), Dravet syndrome (Carvill et al., 2014) and other types of early onset NDD + E (Stamberger et al., 2016). *STXBP1* is also one of most frequently mutated genes in sporadic intellectual disabilities and developmental disorders. All *STXBP1* encephalopathy patients show ID and 95% have epilepsy. *STXBP1* encephalopathy is mostly caused by haploinsufficiency as more than 60% of reported mutations are either deletions, nonsense, frameshift or splice site variants (Stamberger et al., 2016).

Synapsins are a family of synaptic vesicle (SV) phosphoproteins that interact with SVs and the actin cytoskeleton to facilitate the pre‐ and post‐docking stages of neurotransmitter release, thus regulating SV trafficking and short‐term plasticity (Cesca, Baldelli, Valtorta, & Benfenati, 2010). Missense and truncation mutations in the *SYN1* and *SYN2* genes encoding Synapsin 1 and Synapsin 2 have both been associated with NDD with or without epilepsy (Fassio et al., 2011; Lignani et al., 2013; Peron, Baratang, Canevini, Campeau, & Vignoli, 2018). Synapsin 1 and Synapsin 2 mediate the synchronous and asynchronous release of GABA at inhibitory synapses, respectively, thus playing a key role in the inhibitory control of network excitability (Forte, Binda, Contestabile, Benfenati, & Baldelli, 2020; Medrihan, Ferrea, Greco, Baldelli, & Benfenati, 2015).

The proline‐rich transmembrane protein 2 (PRRT2) is a neuron‐specific transmembrane protein, which primarily localizes pre‐synaptically, where it associates the t‐SNARE protein SNAP25 and SVs (Stelzl et al., 2005). PRRT2 has recently been identified as a key component of the vesicular release machinery through its interaction with the Ca2 + sensors Syt1/2, which mediates the synchronous release of SVs (Valente et al., 2016). The important role of PRRT2 at synapses implicates it in key developmental processes and *PRRT2* LoF leads to defects in neuronal migration, spinogenesis and synapse formation and maintenance (Liu et al., 2016; Valtorta, Benfenati, Zara, & Meldolesi, 2016). LoF mutations in *PRRT2* are primarily associated with BFNIE early in childhood and paroxysmal kinesigenic dyskinesia (PKD) early in adolescence or both (PKD with infantile convulsions; Valtorta et al., 2016). However, *PRRT2* mutations have also been associated with neurodevelopmental disorders, such as non‐syndromic ID and ASD and NDD + E, such as Dravet syndrome (Ebrahimi‐Fakhari, Saffari, Westenberger, & Klein, 2015). Extensive genetic and phenotypic characterization is still required to establish the boundaries of the broad spectrum of *PRRT2*‐related diseases, which comprise paroxysmal, seizure and behavioural disorders. However, establishing clear genotype–phenotype correlations will likely be difficult as family members carrying identical *PRRT2* mutations often exhibit variable phenotypes (Brueckner et al., 2014).

While mutations in genes mediating SV release by exocytosis have been widely associated with NDD + E, mutations affecting endocytosis can also be pathogenic (Bonnycastle, Davenport, & Cousin, 2020). Dynamin 1 is a GTPase specifically expressed in neurons, which is involved in SV fission during endocytic processes (Ferguson et al., 2007). Dynamin 1 has been particularly studied for its role in clathrin‐mediated endocytosis, a fundamental process that allows SV recycling after neurotransmitter release at the pre‐synaptic plasma membrane. As clathrin forms an invaginated endocytic bud, dynamin slowly accumulates at the bud neck to tighten it and allow its endocytic release (Cheung & Cousin, 2019; Ferguson & De Camilli, 2012). De novo mutations in the *DNM1* gene, encoding dynamin 1, have been associated with NDD + E in large‐scale genetic studies (Allen et al., 2013). A recent study aimed at characterizing the spectrum of disorders caused by mutations in *DNM1* found that patients show a remarkably homogeneous phenotype, most often displaying severe to profound intellectual disability, hypotonia and refractory epilepsy (von Spiczak et al., 2017). Interestingly, all mutations were clustered in the GTPase or middle domain of the protein and one third of patients had the same recurrent c.709C>T (p.Arg237Trp) mutation. Structural modelling and experimental evidence suggest that the mutations exert a dominant‐negative effect by preventing either the assembly of dynamin oligomers or vesicle scission, thus impairing SV endocytosis (von Spiczak et al., 2017).

#### 1.3.2 Post‐synaptic terminal

GABA_A_ receptors are ligand‐gated ion channels that are the primary mediators of fast inhibitory synaptic transmission in the CNS. GABA_A_ receptors are pentamers formed of different subunits (α1‐ α6, β1‐β3, γ1‐γ3, δ, ɛ, π, θ and ρ1‐ρ3) that assemble to form Cl^‐^ ion channels (Macdonald & Olsen, 1994). Most GABA_A_ receptors are thought to contain two α subunits, two β subunits and one γ or one δ subunit. GABA_A_ receptors mediate both phasic synaptic and tonic peri‐synaptic or extra‐synaptic inhibition and several anti‐epileptic drugs such as benzodiazepines and barbiturates act by enhancing GABA_A_ receptor currents (Macdonald, Kang, & Gallagher, 2012). Knowing the crucial role of GABA_A_ receptor in network inhibition, it is unsurprising that LoF mutations in genes encoding GABA_A_ receptor subunits are associated with severe epilepsy. Missense mutations in *GABRG2*, encoding the GABA_A_ receptor γ2 subunit have been associated with febrile seizures with or without absence epilepsy and GEFS+, while more severe truncation mutations have been associated with a GEFS+/DS phenotype (Harkin, Bowser, & Dibbens, 2002; Kananura et al., 2002). Moreover, deletion and frameshift mutation in the GABA_A_ receptor α1 are associated with DS and juvenile myoclonic epilepsy (JME; Cossette et al., 2002; Steel, Symonds, Zuberi, & Brunklaus, 2017).

AMPA receptors (AMPARs) are tetrameric ligand‐gated ionotropic glutamatergic channels that mediate the fast component of excitatory transmission (Traynelis et al., 2010). *GRIA2* encodes for the GluA2 subunit of AMPARs, which is particularly important as it regulates Ca^2+^ permeability and voltage rectification (Isaac, Ashby, & McBain, 2007). LoF variants in *GRIA2* have been associated with NDD + E (Salpietro et al., 2019). *GRIA2* patients can display a spectrum of phenotypes including, ID, developmental delay or regression, ASD, speech impairment and seizures, notably rolandic spikes (Salpietro et al., 2019).

NMDA receptors are ligand‐gated ionotropic glutamatergic channels that mediate a Ca^2+^‐permeability, slow component of synaptic current, which plays key roles in formation and maturation of excitatory synapses and circuits (Traynelis et al., 2010). The *GRIN* gene family encodes three classes of NMDA receptor (NMDAR) subunits: the glycine‐binding GluN1, glutamate‐binding GluN2 and the glycine‐binding GluN3. Most NMDARs are tetrameric assemblies of two GluN1 and two GluN2 subunits. Mutations in the *GRIN1*, *GRIN2A* and *GRIN2B* genes encoding the GluN1, GluN2A and GluN2B NMDAR have been shown to cause NDDs such as ID and ASD and NDD + E (XiangWei, Jiang, & Yuan, 2018). Recently, de novo heterozygous missense mutations in *GRIN1*, likely resulting in LoF, were found in patients presenting severe ID, movement disorders and seizures (Lemke et al., 2016). Variants in *GRIN2A* and *GRIN2B* account for the majority of reported disease‐causing variants. De novo variants in GRIN2A are predominantly associated with NDD + E and notably the epilepsy‐aphasia spectrum (EAS; Carvill et al., 2013). Interestingly, these can be both missense variants with putative GoF or LoF truncating variants (Myers et al., 2019). The mechanisms by which *GRIN2A* haploinsufficiency could promote hyperexcitability are not yet known. On the other hand, *GRIN2B* is predominantly associated with NDDs such as ID and ASD, but it has also been involved in NDD + E such as Lennox‐Gastaut syndrome and West syndrome (Allen et al., 2013; Myers et al., 2019). Interestingly, a recent functional study found that missense mutations found in Lennox‐Gastaut syndrome and ID patients had a LoF effect, while those found in West syndrome patients had a GoF effect (Fedele et al., 2018). Thus, while LoF in *GRIN2A* appears strongly associated with seizures, the relationship between *GRIN2B* and seizures is more complex.

*SYNGAP1* encodes the synaptic Ras‐GTPase‐activating protein SynGAP, expressed mainly at the synapses of excitatory neurons. SynGAP is a key mediator in the NMDA receptor‐activated RAS‐signalling cascade at the post‐synaptic density, regulating the formation, development and maturation of dendritic spines (Jeyabalan & Clement, 2016). *De novo* nonsense variants in *SYNGAP1* resulting in haploinsufficiency lead to a form of ID with epilepsy, termed MRD5. SynGAP LoF was found to have major consequences for neuronal homeostasis and development, impairing learning and memory (Jeyabalan & Clement, 2016).

Cyclin‐dependent kinase‐like 5 (CDKL5) is a serine threonine kinase, which localizes at the nuclei and dendrites of neurons and plays pleiotropic roles in cell proliferation, neuronal migration, axonal outgrowth, dendritic morphogenesis and synapse formation and maintenance (Zhu & Xiong, 2019). Notably, CDKL5 is important to ensure excitatory synapse stability at post‐synaptic terminals (Ricciardi et al., 2012). Pathogenic LoF variants in *CDKL5* cause CDKL5‐deficiency disorder (CDD), an X‐linked disorder primarily affecting females and characterized by early onset refractory epilepsy, hypotonia, developmental delay, intellectual disability and visual impairment (Olson et al., 2019). In many aspects, the phenotypic spectrum of CDD resembles Rett syndrome (RTT) and it used to be considered an ‘early seizure variant’ of RTT (Mari et al., 2005). However, while it was found that CDKL5 belongs to the same molecular pathway as MECP2, the disease‐causing gene in RTT, CDD is now recognized as a separate clinical entity (Fehr et al., 2013).

### 1.4 Pre‐clinical models of NDD + E

Genetically relevant disease models that faithfully recapitulate fundamental aspects of NDD + E pathophysiology and phenotypes are crucial for developing and testing novel gene therapies pre‐clinically. Recent advances in genome editing have facilitated the generation of transgenic animal models, which have greatly improved our understanding of mechanisms behind NDD + E pathogenesis (Doudna, 2020; Gonzalez‐Sulser, 2020). By mimicking mutations found in patients, these models often reveal key features of NDD + E and are now routinely used to test the safety and efficacy of gene therapies. However, animal models cannot recapitulate all aspects of human development, genetics and pathology, and often fail to present the full spectrum of NDD + E phenotypes (Fallah & Eubanks, 2020; Gonzalez‐Sulser, 2020; Won, Huang, Opland, Hartl, & Geschwind, 2019; Zhao & Bhattacharyya, 2018). Human in vitro models of disease offer the exciting possibility of recapitulating human‐specific features of neurodevelopment, further improving our understanding of NDD + E and our ability to effectively translate therapeutics into the clinic (Amin & Pasca, 2018; Tidball & Parent, 2016; Table 1).

**Table 1:** Mouse and human stem cell models for NDD + E

Gene	Disease	Genotype			Phenotype		
		Patients	Mouse models	Human stem cell models	Patients	Mouse models	Human stem cell models
*MECP2*	Rett Syndrome	X‐linked, LoF mutations (Amir et al., 1999)	Exon 3–4 deletion (Guy et al., 2001), Exon 3 deletion (Chen, Akbarian, Tudor, & Jaenisch, 2001), R168X (Lawson‐Yuen et al., 2007), R255X, T158A (Goffin et al., 2011), T158M, Y120D (Gandaglia et al., 2019), R306C (Lyst et al., 2013)	R306C, 1155del32, Q244X and T158M (Marchetto et al., 2010), exon 3–4 deletion (Cheung et al., 2011), R294X, T158M, V247X and R306C (Ananiev, Williams, Li, & Chang, 2011) (Williams et al., 2014)	Developmental arrest or regression (>1y), deterioration in communication, social and fine motor skills (Singer & Naidu, 2001). Epilepsy in 50%–90% of patients, heterogeneous seizure phenotype with generalized or focal onset (Tarquinio et al., 2017)	Motor and sensory impairments, behavioural dysfunction, myoclonic jerks, spontaneous or handling‐induced seizures (Katz et al., 2012)	Reduced soma size, neurite outgrowth and glutamatergic synapses. Decreased frequency of spontaneous post‐synaptic currents. Adverse effect of RTT astrocytes on WT neurons. (Marchetto et al., 2010; Cunningham Williams et al., 2014)
*FMR1*	Fragile X	X‐linked, >200 CGG expansion, *FMR1* methylation and transcriptional silencing, LoF (Richter et al., 2015)	PTC in exon 5 (Bakker et al., 1994), exon 1 deletion (Mientjes et al., 2006); CGG(98) (Bontekoe et al., 2001), CGG‐CCG (120) (Entezam et al., 2007)	450 CGG repeats (Urbach et al., 2010), 94 CGG repeats (Liu et al., 2012)	ASD, ID and epilepsy in around 20% of cases (Musumeci et al., 1999)	Audiogenic seizures, network hyperactivity, behavioural abnormalities, cognitive deficits. No spontaneous seizures. (Dahlhaus, 2018)	Decreased neurite outgrowth and synapse formation. Increased amplitude and frequency of calcium transients. (Liu et al., 2012)
*UBE3A*	Angelman Syndrome	LoF of maternal allele (Sell & Margolis, 2015)	Maternal exon 5 deletion (Jiang et al., 1998), PTC in exon 5 (Wang, van Woerden, Elgersma, & Borst, 2017)	5bp deletion in exon 6 (Sun et al., 2019)	Microcephaly, ataxia, hypotonia and motor delay. Seizures in 90% of cases, no distinctive seizure phenotype (Buiting et al., 2016).	Mild cognitive impairment, motor and behavioural dysfunction, audiogenic and flurothyl‐induced seizures, abnormal EEG. No spontaneous seizures. (Rotaru et al., 2020)	Altered excitability, increased fAHP, augmented BK channel expression and synchronous, epileptiform activity (Sun et al., 2019).
*TSC1*	Tuberous sclerosis complex	Germline LoF mutations, second‐hit somatic mutation (Curatolo et al., 2008)	*Tsc1^+/‐^* exon 6–8 deletion (Goorden et al., 2007)	Patient‐derived iPSCs: W750X (Nadadhur et al., 2019)	ASD, ID and intractable epilepsy. Tumours: subependymal nodules and subependymal giant cell astrocytomas. Cortical tubers. Less severe than TSC2 mutation.	Cognitive deficits and impairments in social behaviour. No cerebral lesions, no seizures (Goorden et al., 2007)	Increased network activity, hypertrophy, increased OL proliferation and decreased maturation (Nadadhur et al., 2019)
*TSC2*	Tuberous sclerosis complex	Germline LoF mutations, second‐hit somatic mutation (Curatolo et al., 2008)	*Tsc2^+/−^* exon 2 deletion (Ehninger et al., 2008)	Patient‐derived iPSCs: c.1444 − 2A> C (Li et al., 2017), H522T (Nadadhur et al., 2019), N1515 del1573 and Q794X (Zucco et al., 2018) Genetically engineered hESCs: exon 11 deletion (Costa et al., 2016)	ASD, ID and intractable epilepsy. Tumours: subependymal nodules and subependymal giant cell astrocytomas. Cortical tubers. More severe than TSC1 mutation.	Cognitive deficits. No cerebral lesions, no seizures (Ehninger et al., 2008)	Altered synaptic transmission and differentiation in heterozygotes (Costa et al., 2016)
*SCN1A*	FS, GEFS+, Dravet syndrome	LoF mutations, happloinsufficiency (Catterall et al., 2010)	Exon 1 deletion (Miller et al., 2014), Exon 26 deletion (Yu et al., 2006), R1407X (Ogiwara et al., 2007), R1648H ((Escayg et al., 2000), E1099X (Tsai et al., 2015) and A1783V (Ricobaraza et al., 2019)	S1328P (Sun et al., 2016), R1645X (Higurashi et al., 2013), G1410W and I1183CfsX21 (Kim et al., 2018)	Phenotypic severity correlated with genotype, from FS to DS. DS characterized by prolonged febrile and afebrile, generalized clonic or hemiclonic seizures in the first year of life. Cognitive, behavioural and motor impairments in the second year of life. (Catterall et al., 2010)	Ataxia, spontaneous and thermal seizures, premature death, cognitive impairment, behavioural disturbances. Phenotype largely dependent on background strain. (Mistry et al., 2014; Ricobaraza et al., 2019; Yu et al., 2006)	Decreased sodium current densities and action potential firing in inhibitory neurons but not excitatory neurons (Sun et al., 2016).
*SCN2A*	SCN2A encephalopathy	GoF and LoF mutations (Brunklaus et al., 2020)	*Scn2a^Q54^* (Kearney et al., 2001); exon 1 deletion (Planells‐Cases et al., 2001)		SCN2A GoF: neonatal and early onset epilepsy. SCN2A LoF: later‐onset epilepsy, ASD, ID. Both: Variable seizure phenotype but no absence seizures. (Wolff et al., 2017)	Scn2a GoF: focal and absence‐like seizures, stereotyped repetitive behaviour. (Kearney et al., 2001) Scn2a LoF: hyperactivity, anxiety, social and communication impairments, cognitive deficits, absence‐like seizures. (Lena & Mantegazza, 2019)	
*SCN8A*	SCN8A encephalopathy	GoF and LoF mutations (Brunklaus et al., 2020)	N1768D (Veeramah et al., 2012), R1872W (Bunton‐Stasyshyn et al., 2019)		SCN8A GoF: neonatal and early onset epilepsy. SCN8A LoF: ID and myoclonus without epilepsy (Wagnon et al., 2018) Both: variable seizure phenotype, ataxia, dystonia, hypotonia (Larsen et al., 2015)	Scn8a GoF: Spontaneous seizures, premature death. (Veeramah et al., 2012; Stasyshyn et al., 2019)	
*KCNQ2/KCNQ3*	Benign familial neonatal epilepsy	LoF mutations (Shellhaas et al., 2017)	Kcnq2 exon 3–5 deletion (Watanabe et al., 2000), Kcnq2 A306T and Kcnq3 G311V (Singh et al., 2008)		Early onset epilepsy, developmental delay, hypotonia, dystonia (McTague et al., 2016)	Increased sensitivity to PTZ and electrically induced seizures, early onset spontaneous generalized seizures in homozygous but not heterozygous mice. (Singh et al., 2008)	
*KCNT1*	Epilepsy of infancy with migrating focal seizures	GoF mutations (McTague et al., 2013)	P924L (Burbano et al., 2018)		Early onset epilepsy, nocturnal or migrating focal seizures, hypotonia, microcephaly (McTague et al., 2013)	Increased sensitivity to thermal and chemically induced seizures in heterozygotes. Spontaneous seizures, behavioural deficits and decreased lifespan in homozygotes. (Burbano et al., 2018)	
*KCNC1*	KCNC1 encephalopathy	LoF and dominant‐negative mutations (Cameron et al., 2019; Park et al., 2019)			Epilepsy with myoclonic seizures, developmental delay (Park et al., 2019; Cameron et al., 2019)		
*STXBP1*	STXBP1 encephalopathy	LoF mutations, happloinsufficiency (Stamberger et al., 2016)	Exon 3 deletion (Miyamoto et al., 2017), exon 2–6 deletion (Kovacevic et al., 2018)		ID and epilepsy in 95% of cases (Stamberger et al., 2016)	Normal motor function and diurnal behaviour. Myoclonic jerks, spike‐wave discharges, cognitive deficits, hyperactivity, anxiety and altered social behaviour. (Kovacevic et al., 2018)	
*SYN1/SYN2*	ASD, X‐linked focal epilepsy	LoF mutations (Fassio et al., 2011; Corradi et al., 2014)	SynI exon 1 deletion (Chin, Li, Ferreira, Kosik, & Greengard, 1995), SynII exon 9–10 deletion (Rosahl et al., 1995)		ASD and partial epilepsy (Fassio et al., 2011; Corradi et al., 2014)	Spontaneous seizures in homozygous but not heterozygous mice. Autistic‐like social and behavioural alterations in homozygous mice. (Greco et al., 2013; Michetti, Castroflorio, et al., 2017)	
*PRRT2*	Benign familial neonatal epilepsy, Paroxysmal kinesigenic dyskinesia	LoF mutations (Ebrahimi‐Fakhari et al., 2015)	Exon 1–2 deletion (Michetti, Castroflorio, et al., 2017)	c.649dupC (Fruscione et al., 2018)	Early onset epilepsy, dyskinesia, ID and ASD (Ebrahimi‐Fakhari et al., 2015)	Abnormal motor behaviours and motor paroxysms. No cognitive defects, no spontaneous seizures. (Michetti, Castroflorio, et al., 2017)	Increased Na + currents, increased AIS length and augmented spontaneous and evoked activity (Fruscione et al., 2018).
*DNM1*	DNM1 encephalopathy	Dominant‐negative mutations (Spiczak et al., 2017)	A408T (Boumil et al., 2010)		Refractory epilepsy, ID and hypotonia (Spiczak et al., 2017)	Handling and electrically induced seizures in heterozygotes. Spontaneous seizures, ataxia, vision and hearing impairments in homozygotes. (Boumil et al., 2010)	
*GABRG2*	GEFS+, Dravet syndrome	LoF and dominant‐negative mutations (Kananura et al., 2002)	Q390X (Kang et al., 2015)		DS‐like early onset refractory epilepsy, developmental delay (Kananura et al., 2002)	Spontaneous and thermal seizures, premature death. (Kang et al., 2015)	
*GABRA1*	Dravet syndrome, Juvenile myoclonic epilepsy	LoF mutations (Cossette et al., 2002)	Exon 9 deletion (Arain et al., 2012)		Variable seizure type: myoclonus, absence, generalized and focal (Steel et al., 2017)	Absence‐like seizures, premature death. (Arain et al., 2012)	
*GRIA2*	NDD with or without epilepsy, Idiopathic focal epilepsy	LoF mutations (Lemke et al., 2013; Salpietro et al., 2019)			ASD and ID with or without epilepsy. If epilepsy, usually tonic‐clonic, focal or focal with rolandic spikes (Lemke et al., 2013; Salpietro et al., 2019)		
*GRIN1*	GRIN1 encephalopathy	LoF mutations (Lemke et al., 2016)	Nr1‐neo in intron 20 (Mohn, Gainetdinov, Caron, & Koller, 1999), exon 5 deletion (Liu et al., 2019)		Epilepsy, ID and movement disorders (Lemke et al., 2016)	Defects in sensorimotor gating and cognitive deficits in hypomorph mice. Increased susceptibility to chemically induced seizures in homozygous mice but no spontaneous seizures. (Barkus et al., 2012; Liu et al., 2019)	
GRIN2A	Epilepsy and ID, Epilepsy‐aphasia spectrum	GoF or LoF mutations (Myers et al., 2019)	Grin2a KO (Salmi et al., 2018)		Epilepsy, ID, ASD, aphasia, hypotonia, dystonia (Myers et al., 2019)	Epileptiform discharges, no spontaneous seizures. (Salmi et al., 2018)	
*GRIN2B*	NDD with or without epilepsy	GoF or LoF mutations (Fedele et al., 2018)			Epilepsy, ID, ASD (Myers et al., 2019)		
*SYNGAP1*	MRD5	LoF mutations, happloinsufficiency (Deciphering Developmental Disorders Study, 2017)	Exon 7–8 deletion (Kim, Lee, Takamiya, & Huganir, 2003), exon 4–9 deletion (Vazquez, Chen, Sokolova, Knuesel, & Kennedy, 2004), stop codon at exon 4 (Komiyama et al., 2002).		Epilepsy and ID (Deciphering Developmental Disorders Study, 2017)	Cognitive deficits, spontaneous interictal activity and increased sensitivity to fluorothyl‐induced seizures in heterozygous mice. No spontaneous seizures. (Kilinc et al., 2018)	
*CDKL5*	CDKL5 deficiency disorder	X‐linked, LoF mutations (Olson et al., 2019)	Exon 2 deletion (Okuda et al., 2017), conditional exon 4 deletion (Amendola et al., 2014), exon 7 deletion (Wang et al., 2012)	Patient‐derived iPSCs: R59X and L220P (Ricciardi et al., 2012), G347X and T288I (Livide et al., 2015)	Epilepsy, ID, developmental delay, hypotonia, visual impairment. Primarily affects females (Olson et al., 2019).	Hyperexcitability, autistic‐like phenotype, impaired vision, motor control and memory. Reduced dendritic arborization of cortical neurons. Abnormal EEG and increased susceptibility to NMDA‐induced seizures but no spontaneous seizures. (Amendola et al., 2014; Wang et al., 2012; Okuda et al., 2017)	Decreased synapse formation and abnormal spine morphology. (Ricciardi et al., 2012)

ASD, autism spectrum disorder; FS, febrile seizure; GEFS, generalized epilepsy with febrile seizures plus; ID, intellectual disability; PRRT2, proline‐rich transmembrane protein 2.

### 1.5 Animal models of NDD + E

The generation and characterization of NDD + E animal models has been crucial to elucidating pathological mechanisms and testing novel therapies pre‐clinically in vivo.

Many drosophila and zebrafish transgenic models of neurodevelopmental disorders and epilepsy have been generated that recapitulate the aspects of human pathology (Bellosta & Soldano, 2019; Griffin et al., 2018; Vaz, Hofmeister, & Lindstrand, 2019). These models have supported considerable advances in our understanding of the underlying pathophysiology of NDD + E and zebrafish models have been used as a high‐throughput screening tool for novel AEDs. However, many differences in neurodevelopment prevent direct translation between models in non‐mammalian species and human patients. Notably, invertebrate species lack the formation of a laminated cortex, a central part of NDD + E (Griffin et al., 2018). Important genetic and structural differences in neurodevelopment between non‐mammalian models and humans mean that many molecular, cellular, behavioural and network changes associated with NDD + E cannot be directly compared, but instead rely upon careful interpretation of cellular and behavioural readouts (Praschberger et al., 2017).

Although still possessing many limitations in phenotype, rodent models are more genetically and developmentally similar to humans, making them the current model of choice for testing gene therapies in vivo. Notably, recent advances in gene editing using CRISPR‐Cas9 has allowed the rapid generation of many transgenic mouse models for NDD + E available for pre‐clinical investigation. For mouse models to be the most effective medium of clinical translation, it is important that they display both construct validity (similarity at the genotypic level) and face validity (similarity at the symptomatic level). Generating knock‐in mouse models with mutations observed in patients in the best way to ensure construct validity and careful analysis of behavioural and seizure phenotypes is required to demonstrate face validity (Fallah & Eubanks, 2020; Gonzalez‐Sulser, 2020; Katz et al., 2012; Silverman & Ellegood, 2018).

### 1.6 Models of mutations that cause NDD + E through changes in gene expression

These models are less mechanistically direct than changes in ion channels or synaptic proteins which can be directly linked to altered neuronal activity. Thus, it may not be surprising that some of the models that reproduce changes in regulatory genes associated with NDD + E can have variable similarity to the effects of human mutations.

For modelling Rett syndrome, several mouse models expressing different types of *MECP2* mutations are currently available, including truncated (*Mecp2* R168X and R255X) and missense mutations (*Mecp2* T158A, T158M, Y120D and R306C) found in patients (Bertoldi et al., 2019; Gandaglia et al., 2019; Goffin et al., 2011; Guy, Hendrich, Holmes, Martin, & Bird, 2001; Katz et al., 2012; Lyst et al., 2013; McLeod et al., 2013). *Mecp2* mouse models exhibit a broad phenotypic spectrum that resembles Rett syndrome, including motor and sensory impairments, behavioural dysfunction, myoclonic jerks and spontaneous or handling‐induced seizures (Fallah & Eubanks, 2020; Katz et al., 2012).

The first mouse model of FXS was generated via *Fmr1* KO and completely lacks Fmrp expression. However, in humans FXS results from X‐linked inactivation of the *Fmr1* gene and thus, patients still express *FMR1* until at least the 10th week of gestation. To overcome those limitations and achieve better construct validity, several CGG repeat knock‐in mice were generated (Baskaran et al., 2002; Bontekoe et al., 2001; Entezam et al., 2007). Both knock‐in models show decreased FMRP expression but to varying degrees depending on the brain region. However, unlike FXS patients, none of these mice models have reliably shown hypermethylation of the inserted CGG repeats, indicating that the reduced expression of FMRP occurs through a different mechanism in mouse and humans (Brouwer et al., 2008; Entezam et al., 2007). Audiogenic but not spontaneous seizures have been reported in FXS mouse models; however, EEG recordings do suggest network hyperexcitability (Lovelace, Ethell, Binder, & Razak, 2018). *Fmr1* KO mice display a range of behavioural abnormalities such as hyperarousal, anxiety, impaired social interaction and decreased nest‐building or marble‐burying behaviour as well as cognitive deficits (Dahlhaus, 2018).

Transgenic mice recapitulating the genotype of AS with loss of the expressed maternal allele, while the paternal allele silenced by imprinting is preserved (*Ube3a* ^*m‐/p+*^), are currently available (Rotaru, Mientjes, & Elgersma, 2020). They capture many key neurological features of the disorder such as motor deficits, abnormal EEG, anxiety and audiogenic or flurothyl‐induced seizures, but only display mild cognitive deficits and no spontaneous seizures (Rotaru et al., 2020).

Heterozygous TSC mouse models have subtle NDD phenotypes such as cognitive deficits and social impairments but do not show obvious TSC lesions or seizures (Ehninger et al., 2008; Goorden, van Woerden, van der Weerd, Cheadle, & Elgersma, 2007). As homozygosity is embryonic lethal, conditional KO using floxed alleles is necessary to get a more severe phenotype similar to patients (Sahin et al., 2016).

### 1.7 Models replicating mutations in ion channel genes

There are increasing numbers of mouse models available that replicate the *SCN1A* LoF mutations observed in DS. These lines include targeted deletion of *Scn1a* exon 1 and exon 26, as well as knock‐ins of missense and truncation mutations observed in patients: *Scn1a* R1407X, R1648H, E1099X and A1783V (Martin et al., 2010; Miller, Hawkins, McCollom, & Kearney, 2014; Ogiwara et al., 2007; Ricobaraza et al., 2019; Tsai et al., 2015; Yu et al., 2006). *Scn1a+/−* mice typically display spontaneous and hyperthermia‐induced seizures, with ictal and interictal epileptiform activity. The recently developed *Scn1a* A1783V mouse model also displays cognitive impairment, anxiety and hyperactive behaviours, recapitulating the full spectrum of DS phenotypes (Ricobaraza et al., 2019). However, phenotypic severity and survival are highly dependent upon background strain, owing to the presence of strain‐specific genetic modifiers (Miller et al., 2014; Mistry et al., 2014; Mulligan et al., 2019).

Mouse models modelling both LoF and GoF mutations in *SCN2A* have been produced (Hedrich, Lauxmann, & Lerche, 2019). *Scn2a* KO mice modelling haploinsufficiency were found to display a spectrum of autistic‐like phenotypes such as hyperactivity, anxiety, impaired social and communicative behaviour, as well as cognitive deficits (Lena & Mantegazza, 2019; Middleton et al., 2018; Spratt et al., 2019; Tatsukawa et al., 2019). Moreover, conditional KO of *Scn2a* in forebrain excitatory neurons exhibit absence‐like seizures associated with spike‐wave discharges (Ogiwara et al., 2018). The transgenic *Scn2a^Q54^* GoF mice exhibits partial and absence‐like seizures with onset at 2 months, as well as stereotyped repetitive behaviours (Kearney et al., 2006). However, while patients with *SCN2A* encephalopathy do display various types of focal seizures, absence‐like seizures are not commonly reported, suggesting a possible species‐specific effect (Hedrich et al., 2019; Howell et al., 2015).

Two knock‐in mouse models of *SCN8A* encephalopathy have been generated by inserting the less severe Scn8a N1768D and more severe R1872W GoF mutations that are observed in patients. Mice were found to be highly sensitive to GoF mutations in *Scn8a*, N1768D mouse display spontaneous seizures beginning at 2 months of age and premature death subsequently occurring within 1 to 4 weeks. R1872W mice have a more severe phenotype with spontaneous seizures starting at 14 days of age and premature death occurring within 24h (Bunton‐Stasyshyn et al., 2019; Veeramah et al., 2012).

A heterozygous KO mouse model of *KCNQ2* was first generated in 2000 and showed increased sensitivity to pentylenetetrazole (PTZ)‐induced seizures but not spontaneous seizures (Watanabe et al., 2000). Later on, *KCNQ2* and *KCNQ3* LoF mutations observed in BFNIE patients were knocked‐in in mice to develop two new mouse models (Singh et al., 2008). Heterozygous knock‐in mice exhibited reduced threshold to electrically induced seizures, while homozygous knock‐in showed early onset spontaneous generalized tonic‐clonic seizures as observed in patients.

The P924L GoF mutation in *KCNT1*, which has been observed in two patients with EIMFS has recently been knocked‐in in a mouse model. Heterozygous mice did not show any increase in susceptibility to thermal or chemically induced seizures and locomotor activity was normal. However, homozygous mice did display spontaneous seizures, behavioural deficits and decreased lifespan (Burbano et al., 2018).

### 1.8 Models of mutations in synaptic proteins

Different *Stxbp1* ^*+/−*^ mutant mice have been generated that recapitulate disease‐causing *STXBP1* haploinsufficiency in humans (Chen et al., 2020; Kovacevic et al., 2018; Miyamoto et al., 2017; Orock, Logan, & Deak, 2018). *Stxbp1* ^*+/−*^ mice show myoclonic jerks, spike‐wave discharges, impaired cognitive performance, hyperactivity and anxiety with altered social behaviour. Interestingly, deleting *Stxbp1* specifically in GABAergic interneurons leads to early lethality, suggesting a critical role for *Stxbp1* in inhibitory neurons (Kovacevic et al., 2018). Further studies showed that *Stxbp1^+/−^* mice exhibit reduction in the strength of PV+ interneuron synapses likely as a result of a decrease in the number of readily releasable vesicles or release probability, as well as a reduction in the connectivity of SST+ interneurons onto pyramidal neurons, resulting in a decrease in inhibitory inputs (Chen et al., 2020).

Mice constitutively lacking SynI, SynII or both (SynKO) consistently show an impairment of inhibitory function and facilitated excitatory transmission (Baldelli, Fassio, Valtorta, & Benfenati, 2007; Chiappalone et al., 2009; Farisello et al., 2013). This excitation/inhibition imbalance is manifested in SynKO mice by an overt epileptic phenotype and behavioural disturbances including defects in social interactions suggestive of an ASD phenotype and cognitive impairment (Greco et al., 2013; Michetti, Caruso, et al., 2017).

Contrary to BFNIE or PKD/IC patients with *PRRT2* mutations, *PRRT2* KO mice do not exhibit spontaneous seizures. However, they do display motor paroxysms such as gait problems and back walking, which recapitulate the common PKD phenotype observed in *PRRT2* patients (Michetti, Castroflorio, et al., 2017).

Conversely, for dynamin, models were identified by phenotype, when mutant ‘fitful’ mice identified through a forward genetics approach exhibited spontaneous seizures from 2–3 months of age, and were found to have a mutation in a highly conserved *DNM1* exon resulting in a dominant‐negative effect (Boumil et al., 2010). Moreover, the mutation resides in one of two alternate isoforms of dynamin 1, as the expression of the mutated isoform is higher later in brain development, thus this may explain the onset of symptoms after maturation. Homozygous fitful mice display an additional ataxic phenotype with hearing and vision impairments as well as lethal seizures (Boumil et al. 2010). Interestingly, the dominant‐negative effect exerted by the *DNM1* fitful mutation is required for the epileptic phenotype as *Dnm1* null mice do not have seizures (Ferguson et al., 2007).

Post‐synaptic receptors have also been modelled in vivo. A mouse model of the GABRG2 Q390X truncation mutation causing DS has also been recently generated (Kang, Shen, Zhou, Xu, & Macdonald, 2015) Using this model, Kang et al. found that the mutation generates a detectable truncated γ2 subunit suggesting it does not undergo nonsense‐mediated mRNA decay and instead accumulates in ER where it exerts a dominant‐negative effect on WT subunit. *Gabrg2^+/Q390X^* knock‐in mice develop severe epilepsy by P19 with tonic‐clonic seizures with interictal epileptiform activity, as well as increased sensitivity to heat‐induced seizures, impaired memory and social behaviour (Warner, Liu, Macdonald, & Kang, 2017). A *Gabra1^+/−^* mouse model of DS also exists where heterozygous mice show significantly elevated incidence of spontaneous death, absence‐like epilepsy phenotype with slow cortical spike and wave discharges (Arain, Boyd, & Gallagher, 2012). However, those mice do not display spontaneous generalized tonic‐clonic seizures as seen in *GABRA1* patients (Steel et al., 2017).

GluN1 hypomorphic mice only retain 5%–10% expression of the obligatory GluN1 NMDA receptor subunit and were initially proposed as a model of schizophrenia due to deficits in sensorimotor gating (Duncan, Moy, Lieberman, & Koller, 2006). But further behavioural tests indicated deficits in a number of cognitive tests, suggesting it could model ID (Barkus, Dawson, Sharp, & Bannerman, 2012). Moreover, homozygous but not heterozygous deletion of *Grin1* exon 5 led to increased susceptibility to chemically induced seizures (Liu et al., 2019). Epileptiform discharges as well as structural alterations in the cortex of *Grin2a* KO mice have been observed, but not spontaneous seizures (Salmi et al., 2018).

*Syngap1* heterozygous KO mice offer both construct and face validity for a form of ID with epilepsy, termed MRD5, with a 50% reduction in SynGAP protein resulting in cognitive deficits, spontaneous interictal activity and decreased seizure threshold (Clement et al., 2012; Ozkan et al., 2014).

*Cdkl5* KO mouse models of CDD have been generated and recapitulate some phenotypic features observed in patients such as hyperexcitability and deficits in social interaction, vision, motor control and memory (Amendola et al., 2014; Okuda et al., 2018; Wang et al., 2012). However, despite the prevalence of seizures in CDD patients, *Cdkl5* KO mice do not display spontaneous ictal or interictal activity, although they are susceptible to NMDA‐induced seizures (Okuda et al., 2018).

In general, while genes involved in human brain disorders are frequently conserved in rodents, their function may not be as critical to the complex behavioural disabilities associated with NDD + E. This is particularly true where the effect of mutations is indirect, that is, via altered gene expression. In heterozygous mice it also seems possible that compensatory mechanisms come into effect which are not present or have less of an impact in humans. Consequently, not all mouse models the phenotypes observed in patients, and frequently mice must be bred to homozygosity before symptoms (e.g. spontaneous seizures) are observed. Mouse models also reveal the strong phenotypic effects of genetic modifiers in particular different strains of mice demonstrate the importance of the genetic background and suggests that many unknown modifying factors may influence NDD + E phenotypes in humans (de Lange et al.., 2020; Mistry et al., 2014). Importantly, in some cases, although early post‐natal epilepsy is developed in animal models of NDD + E, spontaneous seizures tend to disappear later on in adulthood, that is, in *T* *sc1^+/−^* mouse model (Gataullina et al., 2016). This aspect might be due to species‐specific compensations or to differences in the NDD + E pathophysiology. In the *Tsc1* *^+/−^* mouse model the absence of tubers could be a possible explanation of the difference with the human pathology (Gataullina et al., 2016). Nevertheless, the assessment of potential therapies on the early epileptic phenotype is still important to define the feasibility of treatments in young patients and to analyse the impact of these approaches on the development of cognitive defects.

### 1.9 In vitro human models of NDD + E

Recently, researchers have developed human cell models of NDD + E, taking advantage of the opportunities offered by human embryonic (hESC) and human induced pluripotent stem cell (hiPSC) technology (Niu & Parent, 2020). hiPSCs allow to model disease by generating neuronal cells that carry the specific genetic information of the patient and to test gene therapies in the context of the human genome, which could improve translational success (Zhao & Bhattacharyya, 2018). While it is not possible to observe behaviour in vitro, and consequently in vivo models will remain a necessary part of the research pathway, biochemical, morphological and electrophysiological characterizations of hiPSC‐derived neurons and glia can uncover or confirm important pathological mechanisms and targets underlying NDD + E in humans. These models are particularly important where in vivo models reveal species‐specific differences in gene processing or signalling pathways.

#### 1.9.1 Early 2D human stem cell models

Some of the first human cell models of an NDD + E were developed to model DS, and some of the early results point to challenges with using neurons derived from iPSCs. For example, although in mice, DS was clearly associated with loss of excitability in interneurons, early studies using iPSC‐derived neuronal cells suggested a counterintuitive GoF effect of the A5768G, Q1923R and F1415I missense mutations in *SCN1A*, recording increased excitability in both excitatory and inhibitory neurons (Jiao et al., 2013; Liu et al., 2013). However, the short differentiation of these cells (4–8 weeks) models an early phase of maturation in vivo as control hiPSC‐derived neurons only showed short trains of AP, suggesting inherent functional immaturity. When subsequent studies restricted their analysis of electrophysiological recordings to functionally mature neurons or promoted maturity by co‐culturing neurons on a monolayer of rat cortical astrocytes (Higurashi et al., 2013; Sun et al., 2016), they found a clear LoF effect in DS interneurons carrying *SCN1A* S1328P missense and R1645X truncation mutations, with decreased Nav currents and AP firing that was not seen in excitatory neurons. Thus, the identification of cellular phenotypes in hiPSC‐derived neurons needs to be done in functionally mature neurons to identify defects that are relevant to patients.

An additional challenge for human stem cell models is the role of genetic background. Early studies were carried using only one patient and one age‐matched control hiPSC line. Yet, mouse models and families carrying individual mutations have repeatedly confirmed that differences in genetic background contribute to differences in NDD + E severity. While the use of multiple lines of cells can help to clarify the effects of genetic heterogeneity, isogenic pairs are better able to resolve this issue. Isogenic hiPSC pairs are commonly generated using CRISPR‐Cas9 by correcting the disease‐causing mutation in a patient hiPSC line or introducing it in a control line, thus generating pairs of cell lines that are identical in their genetic background and differ only by the presence or absence of a specific mutation (Hockemeyer & Jaenisch, 2016). Comparing isogenic patient and control lines allows the identification of robust phenotypes that are solely dependent on a mutation. Alternatively, it is possible to generate isogenic lines by using mutated and non‐mutated primary cells from mosaic individuals (Maeda et al., 2016).

#### 1.9.2 Mutations affecting gene regulation in 2D hiPSC models

These mutations, which are known to have effects on the regulation of multiple genes, are particularly vulnerable to species differences. Thus hiPSC‐derived models are central to confirming that candidate pathomechanisms identified in mice (or other species) are relevant to humans. However, the indirect effects of the mutations means that alterations in cell migration (for example), which are more challenging to model in vitro, may limit some of the simpler 2D hiPSC models.

hiPSC models of Rett syndrome have been generated and used to test potential treatments directly on patient cells (Marchetto et al., 2010). Rett hiPSC‐derived neurons showed decreases in soma size, neurite outgrowth, glutamatergic synapse formation and spontaneous activity. Moreover, isogenic controls can be derived through X‐chromosome inactivation (Cheung et al., 2011). Importantly, a study found that control neurons co‐cultured with astrocytes derived from Rett syndrome hiPSCs showed similar abnormal morphological phenotypes, highlighting the importance of including glial cells in in vitro disease models (Williams et al., 2014).

Neurons differentiated from a CDD patient hiPSC line harbouring a *CDKL5* mutation displayed reduced synapse formation and increased dendritic spine length (Ricciardi et al., 2012). More recently, a study comparing neurons differentiated from *MECP2* and *CDKL5*‐mutated iPSC lines found a common alteration in the expression of the glutamate D1 receptor GluD1 (Livide et al., 2015). This work allowed to further elucidate the biological basis of the similar phenotypes observed in CDD and RTT patients.

FXS has been modelled using both hESCs, isolated from human embryos identified through pre‐implantation genetic diagnosis, and hiPSCs derived from FXS patients (Urbach, Bar‐Nur, Daley, & Benvenisty, 2010). Interestingly, comparing those cells uncovered differences in *FMR1* gene expression. In FXS hESCs, the *FMR1* gene is initially expressed but becomes transcriptionally silenced upon differentiation, whereas in FXS hiPSCs the *FMR1* locus remains inactive as it is not reset to a transcriptionally active state through reprogramming. However, while FXS hiPSCs do not model the differentiation‐dependent silencing of the FMR1 gene, they remain valuable tools to analyse the role of *FMR1* in neural cells as in both FXS iPSCs and FXS neurons the *FMR1* gene is methylated (Urbach et al., 2010). Neurons derived from FXS hiPSCs show reductions in neurite length and synapse formation as well as an increased amplitude and frequency of calcium transients, suggesting network hyperactivity (Liu et al., 2018).

Dorsal telencephalic neural precursor cells derived from TSC patients with heterozygous *TSC2^+/−^* mutations exhibited increased proliferation rate in some studies (Li et al., 2017) but not others (Zucco et al., 2018). Similarly, studies investigating changes in cellular excitability and network activity showed conflicting findings for *TSC2^+/−^* neurons (Costa et al., 2016; Nadadhur et al., 2019). Like for mouse models, complete loss of *TSC1* or *TSC2* seems to be required to achieve a proper TSC phenotype in excitatory cortical neurons (Afshar Saber & Sahin, 2020). This may be due to the fact that other neuronal cell types are involved in the disease pathogenesis and thus required to recapitulate the disease pathology. Interestingly, heterozygous *TSC1* and *TSC2* mutations in in the oligodendroglial lineage leads to increased oligodendrocytes proliferation and decreased maturation (Nadadhur et al., 2019).

However, while iPSC‐derived neurons grown in 2D have been instrumental to further our understanding of disease mechanisms in humans, they lack many distinguishing characteristics of the developing human brain. For instance, cell–cell and secreted ligand–receptor interactions are crucial for choreographing neurodevelopment and synaptic activity, but those signalling dynamics are hampered when neurons are grown in a monolayer. Moreover, 2D cultures often lack glia and can only be kept for a restricted amount of time, preventing the emergence of important late‐stage developmental properties such as gliogenesis and myelination, as well as further electrophysiological and synaptic maturation. For these reasons, researchers are now looking into developing 3D human models of disease using human brain organoids (Amin & Pasca, 2018).

### 1.10 3D human stem cell models

Cerebral organoids can be generated in an undirected manner, in the absence of inductive cues, resulting in 3D structures comprising cells from multiple brain regions such as the cortex, retina or hindbrain (Lancaster et al., 2013). This protocol has been successfully used to model microcephaly, identifying premature neuronal differentiation as a key pathogenic mechanism. However, as this method relies on the stochastic generation of different neuronal cell types, it has high levels of batch‐to‐batch variability. More recently, researchers have tested specific combinations of morphogens and signalling molecule that can pattern 3D aggregates into specific brain regions, such as the cortex (Mariani et al., 2015; Pasca et al., 2015; Qian et al., 2016). Transcriptomic and epigenomic analyses revealed that human cortical spheroids (hCS) can faithfully recapitulate fetal and early post‐natal human cortical development (Pasca et al., 2015; Trevino et al., 2020). hCS derived from AS patients show signs of epileptogenic activity as evidenced by increased network synchrony in pyramidal neurons, caused by enhanced BK channel activity (Sun et al., 2019). Importantly, those results were also confirmed in an AS mouse model, where using a BK channel antagonist ameliorates seizure susceptibility. This suggests that using human and mouse disease models in combination could be a powerful way of establishing pathological mechanisms and improving our ability to test novel therapeutics pre‐clinically.

hCS have also been used to model TSC, but as for mice and 2D models complete loss of TSC through a double‐hit somatic mutation was required for pathogenesis (Blair, Hockemeyer, & Bateup, 2018). As for 2D models, this may be due to the fact that hCS only contain forebrain excitatory neurons and using organoid models that contain other neuronal cell types such as cortical interneurons may yield different outcomes.

Indeed, as hCS are directed towards a forebrain, pallial fate, they do not contain inhibitory neurons that are generated in the subpallium and migrate tangentially to meet their cortical excitatory partners during development (Wonders & Anderson, 2006). Therefore, in order to investigate the role of GABAergic interneurons in disease, fused human subpallium spheroids (hSS) with hCS to form ‘assembloids’ have been recently implemented (Birey et al., 2017). Remarkably, interneurons generated in the hSS migrate into the hCS and form functional synapses with excitatory neurons, creating cortical microcircuits. A recent study used these models to show a critical and unexpected role for *CACNA1C* in the migration of interneurons (Birey et al., 2017). Cortical organoids can also be used to model neuron–glia interactions during neurodevelopment and disease as they contain astrocytes and a modified protocol can be used to induce the generation of myelinating oligodendrocytes (Marton & Pasca, 2020).

Transgenic mouse models have been instrumental in advancing our understanding of the biological basis of NDD + E and they provide a fundamental platform for the development of novel gene therapies in vivo. However, important species differences in genetics and neurodevelopment between mice and humans prevent some mouse models from fully recapitulating NDD + E phenotypes, and this means studies in mouse models are increasingly being paired with data from human cells. Recent advances in human 2D and 3D neuronal models of disease offer the opportunity to identify human‐specific features of mutations linked to NDD + E and testing gene therapies within a human genomic context. However, human models still have the severe limitations of not recapitulating complete physiological brain structure and not including external stimuli, which are fundamental during development. Therefore, combining in vivo mouse and in vitro human models of NDD + E can be the way forward to shed new light on those disorders and improve our ability to translate novel gene therapies into the clinic.

## 2 Gene therapy for NDD + E

Gene therapy seeks to alleviate diseases by introducing genetic material into target cells to restore physiological functions. For most NDD + E disorders the underlying cells are neurons and therefore physical accessibility creates hurdles. This is because most therapies require CNS delivery, either directly to the brain to bypass the blood–brain barrier (BBB) or systemically with a delivery system able to efficiently target the whole brain.

An additional constraint is that gene therapy for NDD + E may need to be administered within a critical period before mature circuits are in place, possibly even embryonically, to rescue all aspects of the pathogenic phenotype (Wykes & Lignani, 2018) (Table 2). Although this is unconfirmed and may need to be empirically tested for each NDD + E treatment.

**Table 2:** Gene therapies for NDD + E

Disease	Therapy and vector	Delivery	Outcomes	Reference	Major hurdles to gene therapy
Rett syndrome	*MECP2* (AAV9)	Tail vein IV injection, ICV injection	Increased survival and improved behavioural phenotypes.	Gadalla et al., 2013	Over‐supplementation can cause NDD. Mosaicism makes supplementation difficult to target.
	*MECP2* (AAV9)	Tail vein injection, intracranial injection	Increased survival and improved behavioural phenotypes.	Garg et al., 2013	
	Instability‐prone *MECP2* (AAV‐PHP.eb)	Tail vein IV injection	Increased survival and improved behavioural phenotypes.	Luoni et al., 2020	
Fragile X syndrome	*Fmr1* (AAV5 with AAV2 ITR)	IH injection	Rescued hippocampal deficits.	Zeier et al., 2009	Over‐supplementation can cause NDD. Mosaicism makes supplementation difficult to target.
	*Fmr1* (AAV9)	ICV injection	Improved limited behavioural phenotypes.	Golizadeh, et al. 2014	
Angelman syndrome	Cas9 targeting *mGluR5* (Gold nanoparticles)	Intracranial injection	Reduced repetitive behaviours	Lee, Guenther, et al., 2018	Over‐supplementation can cause NDD. Over‐supplementation can cause NDD. Mosaicism makes supplementation difficult to target.
	*UBE3A* (AAV9)	IH injection	Improved seizure, ataxia and growth phenotypes.	Daily et al., 2011	
Tuberous sclerosis complex	*Ube3a*‐ATS antisense oligonucleotide	IC and IH injection	Improved associative learning.	Meng et al., 2015	Precise TSC over‐expression should be pursued. Gene supplementation should ideally target *TSC1* or *TSC2*deficient cells.
	*TSC1* (AAVRH8)	ICV injection	Increased survival and improved motor phenotype and brain pathology	Prabhakar et al., 2015	
*CDKL5*‐deficiency disorder	*TSC1* (AAVRH8 and AAV9)	ICV injection and IV injection	Increased survival at P0 and P21 and improved motor phenotype and brain pathology	Prabhakar et al., 2019	Mosaicism makes gene supplementation difficult to target.
*CDKL5*‐deficiency disorder	*CDKL5* (AAV‐PHP.B)	Intrajugular injection	Improved limited behavioural phenotypes.	Gao et al., 2020	Mosaicism makes gene supplementation difficult to target.
*DNM1* encephalopathy Dravet syndrome	Anti‐*Dnm1* miRNA (AAV9)	ICV injection	Improved seizure, ataxia and growth phenotypes.	Aimiuwu et al., 2020	Gene silencing needs to be pathogenic allele‐specific. *SCN1A* is larger than the AAV packaging limit. Supplementation needs to specifically target interneurons
	Anti‐*Scn1a* antagoNAT	Lumbar intrathecal injection	Improved seizure phenotype.	Hsiao et al., 2016	
	*Scn1b* (AAV9)	ICV and intracisterna magna injection	Sexually divergent rescue of limited phenotypes.	Niibori et al., 2020	
	CRISPRa targeting *Scn1a* (dual AAV9)	ICV injection	Improved febrile seizure phenotype.	Colasante, Qiu, et al., 2020	
*SCN8A* encephalopathy	Anti‐*Scn8a* antisense oligonucleotide	ICV injection	Improved survival and delayed seizure onset	Lenk et al., 2020	*SCN8A* is larger than the AAV packaging limit

CDKL5, Cyclin‐dependent kinase‐like 5; IC, infantile convulsions; ICV, intracerebroventricular; IH, intrahippocampal; MECP2, methyl‐CpG binding protein 2; NDD, neurodevelopmental disorders with epilepsy

### 2.1 Classical gene therapy approaches: Gene replacement or supplementation

Most research on gene therapy for NDD + E has focused on exogenously altering the expression of genes. The most straightforward of these approaches consist of delivering a supplemental transgene to cells lacking that gene in order to restore their healthy function. Alternatively, tools that selectively reduce the expression of target genes may be applied, such as antisense oligonucleotides (ASOs) or RNA interference (RNAi; Rinaldi & Wood, 2018; Setten, Rossi, & Han, 2019). While these methods have had successes and led to clinical trials for a number of diseases (High & Roncarolo, 2019), progress for NDD + E has lagged with therapies only just starting to enter trials (i.e. Stoke Therapeutic and Encoded Therapeutics for Dravet syndrome, or Ultragenyx for Angelman syndrome), suggesting these disorders could benefit from more sophisticated strategies. Additionally, altering the expression levels of genes involved in NDD + E disorders often requires careful dosing because of the essential roles those genes play in nervous system development. Indeed, over‐shooting the required change in gene expression is often associated with its own NDD pathology (Meins et al., 2005; Oostra & Willemsen, 2003). The issue of mosaicism further complicates cellular targeting for many NDD + E disorders caused by X‐linked genes or imprinted alleles such as FXS, RTT and AS.

Genome editing is a promising alternative to gene supplementation as a way to cure NDD + E by restoring the genome to the normal state. The CRISPR/Cas gene editing system has become increasingly prominent in the field of gene therapy in recent years as a programmable form of genome editing (Doudna, 2020). In its most simple application, CRISPR/Cas is commonly used to inactivate genes at the genomic level by introducing insertion and deletion mutations (indels) at sites where it makes double‐stranded breaks (DSBs; Dai et al., 2016; Wang, Zhang, & Gao, 2020). While a homozygous deletion would be predicted to exacerbate most NDD + E, where there are heterozygous dominantly inherited mutations, it may be possible to target the pathogenic allele (Christie et al., 2020; Gao et al., 2018). Alternatively, in a manner similar to more traditional gene therapies, catalytically dead Cas9 (dCas9) proteins have also been adapted with a variety of effector domains such as transcriptional activators (CRISPRa) and repressors (CRISPRi) and can be used to modulate endogenous gene expression while maintaining normal biogenesis (Chavez et al., 2015; Qi et al., 2013). Importantly, these approaches work off the endogenous genes, and allow recapitulation of the complex mRNA transcripts; however, they would still require careful dosing. Yet, for all Cas9‐based approaches, strong concerns remain around the increased risk of off‐target editing from long‐term expression and the potential immunogenic response to a bacterial protein (Wang, Mou, & Li, 2015). While autoimmune CNS disorders associated with antibodies against nuclear neuronal proteins are not common, rare forms of autoimmune encephalitis involve membrane proteins (Platt, Agalliu, & Cutforth, 2017).

There is now a large body of research describing treatments targeting non‐genetic epilepsy (Colasante, Qiu, et al., 2020; Ingusci, Verlengia, Soukupova, Zucchini, & Simonato, 2019; Walker & Kullmann, 2020). These approaches aim to control seizures where there is no underlying genetic cause, and thus are not discussed here where the focus is on NDD + E with known genetic causes.

### 2.2 Gene therapy delivery for NDD + E

Currently the most well‐established delivery vectors for gene therapy are viral, with adeno‐associated virus (AAV) in particular being predominant for in vivo gene therapy (Lykken, Shyng, Edwards, Rozenberg, & Gray, 2018). AAV vectors are single‐stranded DNA parvoviruses with unique features that are desirable for therapy, including low genomic integration, low immunogenicity and natural tissue tropism (Li & Samulski, 2020). For CNS disorders, AAV9 are often the AAV of choice due to their natural tropism towards neuronal transduction and a modest ability to cross the BBB enabling systemic delivery (Manfredsson, Rising, & Mandel, 2009). AAV vectors also support long‐term expression of their payload, which is desirable where the payload is acting as a supplement because it reduces the need for repeat dosing. However, it could be a risk for CRISPR/Cas genome editing, where prolonged expression is associated with an immune response to the bacterial protein and off‐target editing (Merkle et al., 2015).

AAV vectors can be delivered systemically to treat NDD + E or via stereotactic injection to bypass the BBB in vivo. To obtain maximal efficiency or study a specific brain region, most proof of concept studies use stereotactic injections such as the intracranial (IC), intrahippocampal (IH) or intracerebroventricular (ICV) routes. While stereotactic injections are still a viable route for NDD + E patients, systemic routes capable of mediating nervous system gene delivery could be considered more ideal for the clinic as they are less invasive. In humans, this is commonly achieved via intravenous (IV) infusion or intrathecally (IT) for CNS disorders. For rodents, IV injection is the most common systemic route, typically using tail vein, although retro‐orbital and intrajugular (IJ) injections have also been used. With the current AAV vectors, peripheral delivery is relatively inefficient and requires a high dose that can cause significant adverse effects. Improved gene therapy vectors may address this issue (as discussed below).

### 2.3 Current NDD + E gene therapy

Gene therapy has been shown to be effective in a variety of NDD + E animal models, with several strategies moving towards clinical trials. Multiple results in these pre‐clinical studies seem to emphasize the importance that developmental stage of therapy could play in the outcome to the disease phenotype, with earlier intervention providing more complete effects.

### 2.4 Gene therapy for NDD + E associated with altered gene expression

These disorders are natural candidates for gene therapy because the underlying genes control a range of important downstream pathways that are hard to target individually. Several groups are making progress in targeting the causative genes; however, timing and dosage are important concerns.

#### 2.4.1 Rett syndrome

Reactivation of the *Mecp2* gene in RTT mice has been shown to significantly reverse their pathological phenotype (Guy, Gan, Selfridge, Cobb, & Bird, 2007). Based on this observation, several groups have explored *Mecp2* supplementation therapy in animal models of RTT. Systemic intravenous (IV) tail vein infusion of a self‐complementary AAV9 vector delivering the human *MECP2* gene to *Mecp2* null mice is sufficient to extend their lifespan by a median of 5 weeks, rescue motor and behavioural phenotypes and halt disease phenotype progression (Gadalla et al., 2013; Garg et al., 2013). However, *MECP2* duplication is associated with a different NDD (Meins et al., 2005), meaning that expression levels of *MECP2* need to be kept within a narrow window. The amount of over‐expression of *MECP2* required for duplication syndrome‐associated toxicity in mouse models has been reported between 1.6 times normal levels and 2.4 times normal levels (Jugloff et al., 2008; Koerner et al., 2018), suggesting that expression should be kept below double that of healthy individuals. Recently, delivery of an instability‐prone *Mecp2* transgene with inefficient translation was shown to still improve the diseases phenotype in RTT mice while causing a more subtle increase in *Mecp2* expression with potential less associated risk (Luoni et al., 2020).

#### 2.4.2 Fragile X Syndrome

Post‐natal ***Fmr1*** supplementation therapy has shown promise for treating FXS, suggesting that the disease phenotype could at least be partially reversed after birth. AAV delivery of *Fmr1* ameliorates the hippocampal dysfunction and behavioural phenotypes of *Fmr1*‐KO mice (Gholizadeh, Arsenault, Xuan, Pacey, & Hampson, 2014; Zeier et al., 2009). However, while a two‐fold increase in expression is well tolerated in *Fmr1*‐KO mice, a 2.5 or higher fold increase induced motor hyperactivity and suppressed the startle response relative to WT mice (Arsenault et al., 2016), emphasizing the risk associated with *FMR1* replacement. CRISPR has been applied to reactivate *FMR1* transcription as an alternative to supplementation in several different ways in vitro. In a patient‐derived FXS hESC line, CRISPRa was able to transcriptionally reactivate FMR1, but without a corresponding change in FMRP expression (Haenfler et al., 2018). It was posited that this was due to the transcribed CGG repeats preventing FMRP translation, underscoring the potential limitations of using CRISPRa in genes that are normally silenced. In an alternative strategy, CRISPR/Cas9 was used to correct the genetic cause of FXS by excising the expanded CGG repeat in human FXS iPS cells, causing a reactivation in FMRP expression in 20% of isolated colonies (Xie, et al., 2016).

Other therapeutic targets have been suggested for FXS on the principle of rebalancing protein synthesis and synaptic function without directly addressing the change in *FMR1* (Krueger & Bear, 2011), with mGluR5 being among the most studied (Bear, Huber, & Warren, 2004). While pre‐clinical studies on pharmacological inhibition of mGluR5 in FXS mice have had successes (Richter et al., 2015), clinical trials using negative allosteric modulators have failed to date (Berry‐Kravis et al., 2016) possibly due to tolerance being developed, which has been observed in mice (Yan, Rammal, Tranfaglia, & Bauchwitz, 2005). In FXS mice, CRISPR‐mediated knockout of *mGluR5* in the striatum of FXS mice was sufficient to rescue exaggerated repetitive behaviours (Lee, Lee, et al., 2018), raising the potential that gene therapy against *mGluR5* could be successful where drugs have failed. More recently, the GSK3α pathway has been identified as alternative target with a preferable profile of side effects to mGluR5 (McCamphill, Stoppel, Senter, Lewis, & Heynen, 2020).

#### 2.4.3 Angelman syndrome

Because Angelman syndrome is caused by the LoF mutations in maternal *UBE3A*, hippocampal injection of an AAV9 delivering the *UBE3A* gene has been used as a gene therapy in AS mice, where it was found to markedly improve associative learning (Daily et al., 2011). However, *UBE3A* over‐expression is strongly associated with autism spectrum disorders (Vatsa & Jana, 2018), making over‐expression potentially hazardous in humans. The paternal *UBE3A* gene is normally silenced by the *UBE3A* antisense transcript *UBE3A*‐ATS (Meng, Person, & Beaudet, 2012), making the maternal copy the only functionally active allele. Therefore, Meng et al. took an alternative approach to reactivate expression of the paternal *Ube3a* allele in AS mice by using an antisense oligonucleotide (ASO) to silence the murine *Ube3a*‐ATS, enabling more controlled Ube3a expression (Meng et al., 2015). While this was also sufficient to rescue contextual fear learning, it had no effect on behavioural and motor phenotypes.

Reactivation of the *Ube3a* gene in AS mice at different developmental stages highlights critical windows for intervention for aspects of the disease phenotype that may explain the limited success of the aforementioned gene therapies in adult mice. *Ube3a* reinstatement ceases to rescue motor development between 3 and 6 weeks post‐natally while only embryonic reinstatement affects anxiety and ASD behaviours (Silva‐Santos et al., 2015). Additionally, epileptic phenotypes can be corrected at P21 but not adulthood (Gu et al., 2019). This suggests an AS gene therapy that corrects all morbidities could have to be early in development.

#### 2.4.4 Tuberous sclerosis

Hamartin supplementation therapy has been investigated as a potential TSC treatment. For the *Tsc1*‐floxed and stochastic *Tsc1*‐floxed mouse models, ICV injections of an AAVrh8 vector delivering the human *TSC1* gene at P0 was sufficient to rescue brain pathology biomarkers, extend lifespan and improve rotarod performance (Prabhakar et al., 2015, 2019). Remarkably, IV injection of the vector at P21 was also sufficient to extend survival in TSC mice, suggesting that at least certain aspects of the disease can be treated with intervention later in life (Prabhakar et al., 2019). However, the effects of gene supplementation therapy on learning and memory have not been examined.

## 3 GENE THERAPY FOR MUTATIONS AFFECTING ION CHANNELS

In some cases, such as potassium channels, the affected genes can be delivered directly; however for larger genes, such as sodium channels, supplementation in viruses (which are restricted by payload) is not possible. In addition, a large proportion of mutations affecting ion channels result in toxic GoF effects, where supplementation is not appropriate. Gene therapy research for these disorders is consequently not often a straightforward replacement of a mutant gene.

### 3.1 Dravet syndrome

Given that the majority of DS cases are caused by the LoF mutations in *SCN1A*, most attempts at gene therapy strategies focus on increasing Nav1.1 activity. However, this is a difficult scenario for several reasons. For one, because DS seems to be caused by an imbalance in excitation and inhibition related to loss of *SCN1A* expression that predominantly affects interneurons (Yu et al., 2006), it is important to bias the increase in Nav1.1 function towards this population of cells to restore the balance. Additionally, the size of the *SCN1A* gene (6 kb) far exceeds the packaging limit of AAV vectors (~4.5kb).

The first proposed therapeutic approach was based on oligonucleotide‐based compounds (AntagoNATs) targeting an antisense non‐coding RNA which control *Scn1a* expression (Hsiao et al., 2016). This approach was able to up‐regulate *Scn1a* both in vitro and in vivo in a Dravet mouse model as well as in non‐human primates. At the moment the main limitation for this approach may be the AntagoNATs half‐life, requiring weekly intrathecal injections to achieve a constant level of *Scn1a* up‐regulation.

In another attempt to circumvent these barriers, an AAV delivering the gene for the Navβ1 auxiliary subunit under the control of a *Gad‐1*‐derived promoter was administered via ICV injection in *Scn1a^+/−^* mice (Niibori, Lee, Minassian, & Hampson, 2020). While this led to a partial rescue of disease phenotypes that was sexually divergent, the promoter used was only moderately selective for GABAergic neurons which may have hindered its efficacy. Furthermore, Navβ1 interacts with many different sodium channels, and thus may not provide a selective therapy. While this study shows a proof‐of‐principle for treatment, it may not be sufficiently effective or specific to translate to clinic.

Another approach that was more specifically aimed to correct the underlying cellular pathology was developed using CRISPRa to increase endogenous expression of*Scn1a* (Colasante, Lignani, et al., 2020; Yamagata et al., 2020). Using the forebrain interneuron selective mDlx5/6 enhancer to drive dCas9 expression and delivering the necessary components across two AAV9 vectors in neonatal mice, those studies showed a significant reduction of febrile seizures in *Scn1a^+/−^* mice relative to WT controls while restoring *Scn1a* expression to healthy levels (Colasante, Lignani, et al., 2020; Yamagata et al., 2020). However, seizures were not fully suppressed, possibly due to the selective forebrain expression of the mDLX promoter or the limited co‐expression of the two AAVs in interneurons. Seizures were also not completely rescued using transgenic Dravet mice expressing dCAS9‐VPR in VGAT‐positive inhibitory neurons, this is probably due to a late intervention (4‐week‐old animals) and/or to a low transduction efficiency (Colasante, Lignani, et al., 2020; Yamagata et al., 2020). Importantly, these treatments will rely upon long‐term expression of the exogenous Cas9 proteins in neurons which could represent a risk for immunogenicity and/or off‐target effects.

### 3.2 SCN8A and SCN2A encephalopathy

*SCN8A* encephalopathy caused by de novo GoF mutations in Na_v_1.6 results in neuronal hyperactivity. A recent study showed that reducing levels of the *Scn8a* transcript with an antisense oligonucleotide (ASO) is a potential translational therapy strategy. Scn8a reduction of 25% to 50% resulted in delayed seizure onset and prolonged survival not only in a mouse model of *SCN8A* encephalopathy but also in Dravet Syndrome (Lenk et al., 2020). Although at the moment there are no gene therapies for LoF *SCN8A* and *SCN2A* mutations, or for GoF *SCN2A* mutations, the development of new strategies is ongoing. CRISPRa to up‐regulate the decreased channel expression, or ASO to decrease their pathological increase, are among the potential therapeutic options for these pathologies.

### 3.3 Targeting mutations affecting synaptic proteins

In many cases, strategies for targeting synaptic proteins are similar to those used for ion channels. In particular, the CRISPRa‐based approach could translate to any NDD + E diseases caused by genes too large for AAV packaging such as *SYNGAP1*, where re‐expression of the protein in a haploinsufficient mouse model is able to attenuate behavioural phenotypes (Creson et al., 2019), or for *STXBP1* (Stamberger, Weckhuysen, & De Jonghe, 2017). Timing, cell type specificity and dominant‐negative mutations are emerging challenges.

### 3.4 DNM1 epileptic encephalopathy

For *Dnm1* fitful mice, where pathology is caused by a dominant‐negative effect of the mutant allele, *Dnm1* knock‐down based on microRNA delivered by a single bilateral ICV scAAV9 injection during early post‐natal development was sufficient to reduce harmful phenotypes and cellular features (Aimiuwu et al., 2020). Although knock‐down was effective in homozygous *Dnm1* fitful mice, dominant‐negative mutations in humans are usually heterozygous. Therefore, the ideal approach would be to selectively inactivate the mutant allele without affecting the healthy allele and risking an exacerbation of pathology.

### 3.5 CDKL5 deficiency disorder

AAV delivery of the human *CDKL5* has been shown to improve the behavioural phenotype of *CDKL5* knockout mice and restore synaptic effects in neurons derived from iPSCs from *CDKL5*‐deficient patients (Gao et al., 2020). Each isoform of *CDKL5* rescued different parameters in patient‐derived neurons, emphasizing the limitations of exogenous gene supplementation, where endogenous splicing is lost. This suggests a CRISPRa‐based approach may offer improved efficacy as once produced the *CDKL5* transcripts would be processed according to endogenous cellular machinery. Although this paper has several limitations (i.e. no iPSC control lines, no complete recapitulation of the human phenotype and weak effects of the treatments on some parameters), the study is a first proof of concept that may help to establish new therapies and therapeutic strategies for CDKL5.

## 4 New avenues for gene therapy and delivery

### 4.1 Improving AAV vectors

Despite being the foremost delivery vector for NDD + E gene therapy studies, the AAV delivery system has several limitations. Chief among these is the packaging limit (<4.7Kb), which excludes the delivery of many NDD + E genes such as channels. Using CRISPRa and CRISPRi to increase endogenous expression can be used to obviate this packaging limit for larger genes, but in cases where genes are transcriptionally silenced or mutations result in having a dominant‐negative effect, this may not be effective (Haenfler et al., 2018). NDD + E gene therapy also requires CNS delivery and, as discussed, likely needs to be administered early in development to treat all aspects of the disease phenotype before circuit formation is complete. However, even AAV9, one of the most BBB permeable capsids, only crosses the BBB modestly when administered systemically in perinatal and neonatal rodents, non‐human primates and humans (Mattar et al., 2013; Rahim et al., 2011). Stereotactic injection methods are capable of bypassing the BBB, but these are invasive, and only reach a local region of the brain. In the case of NDD + E it is often important to transduce as many CNS cells as possible in order to restore normal circuit formation, making widespread systemic CNS transduction highly desirable. The immune response to AAV vectors is another impediment, both at the level of the immune response and pre‐existing host antibodies to AAV (Rabinowitz, Chan, & Samulski, 2019). When translating AAV gene therapy to humans it is common to encounter problems with efficiency due to these issues, arguing the need for improved delivery vectors. Engineered AAV vectors that selectively improve these features is one approach to creating vectors with better clinical utility (Li & Samulski, 2020). Two main engineering approaches have been used with promising results. Rational design approaches are guided by structure and function, repurposing and altering motifs in ways that generate desired properties (Lee, Guenther, & Suh, 2018). Directed evolution on the other hand uses a selective pressure to screen for candidate modifications that cause desired characteristics (Bartel, Weinstein, & Schaffer, 2012).

Rational design has been used to create AAV vectors with enhanced properties based on insights of how the AAV capsid interacts with its receptors. For example, AAV9.HR is an AAV9 variant containing two modified amino acids that maintains BBB penetrance and neuronal tropism, but transduces peripheral tissue less (Wang et al., 2018). This type of tropism could be ideal in gene therapy for NDD + E caused by genes that are also expressed by peripheral tissue, such as *FMR1*. Rational design has also been used to increase the efficiency of transduction by mutating the AAV9 capsid to evade proteasomal degradation (Petrs‐Silva et al., 2009). Ultimately, rational design can be a powerful tool when building on well‐understood capsid‐receptor interactions, otherwise it has limitations. The crystal structures of most common AAV serotypes have been made available which could facilitate research into the AAV capsid and the development of chimeric AAV serotypes.

Directed evolution can overcome the limitations of rational design by generating delivery vectors that perform well in specific roles without an exact understanding of the underlying mechanisms (Packer & Liu, 2015). Directed evolution can be used to select variants with properties that are desirable for targeting NDD + E but controlled by poorly understood mechanisms, such as high BBB penetrance. Indeed, using a bespoke Cre‐based evolution system called CREATE, an AAV9 variant called AAV‐PHP.B was developed with extremely high BBB penetrance and neuronal transduction efficiency (Deverman et al., 2016). Further evolution of AAV‐PHP.B yielded AAV‐PHP.eB, with enhanced CNS transduction and AAV‐PHP.S, which transduces the PNS efficiently and does not cross the BBB (Chan et al., 2017). AAV‐PHP.eB has been used to improve the disease phenotype in RTT mice via efficient CNS‐wide transduction from a single non‐invasive injection (Luoni et al., 2020), validating the conceptual utility of the AAV‐PHP vectors for NDD + E gene therapy. However, these vectors were raised in C57BL/6J mice, and their CNS tropism is limited in other species and mouse strains, constraining their utility (Hordeaux et al., 2018). This underscores a key limitation to directed evolution; delivery vectors raised against a specific selection pressure can lose their performance when applied to a different biological context. But the discovery that the receptor LY6A is essential for AAV‐PHP transduction could inform production of a variant that is effective in humans (Huang et al., 2019).

Recently, the CREATE system has been developed further into a multiplex system called M‐CREATE to enable identification of variants through multiple selection pressures (Ravindra Kumar et al., 2020). M‐CREATE was used to identify AAV variants that can cross the BBB across murine strains and exhibit tropism for neurons, astrocytes or vascular cells, enabling unprecedented systemic and cell‐specific CNS transduction. In particular, AAV‐PHP.N is a variant with high specificity for neurons and reduced liver transduction, which could allow highly specialized delivery for NDD + E. The capsids identified by M‐CREATE have not yet been tested outside the mice, but it will be valuable to know how well these properties are conserved across species. Understanding the underlying mechanisms of BBB penetrance across these different variants could enable the design of vectors with similar utility in humans, while the data produced by future M‐CREATE experiments can be used to train in silico AAV variant design. Possibly, M‐CREATE could also be used to study AAV variants that can efficiently cross the placenta for non‐invasive perinatal delivery in NDD + E, although making genetic interventions during such a critical stage of development could be a relatively high risk.

### 4.2 Building on the CRISPR toolbox

The CRISPR/Cas genome editing system has been adapted for a wide range of new applications, creating new tools with increased utility and efficiency (Doudna, 2020). The versatility of the system is advancing with a range of natural and synthetic Cas alternatives to WT SpCas9, such as XCas9 and SpRY with non‐conventional PAM recognition (Hu et al., 2018; Walton, Christie, Whittaker, & Kleinstiver, 2020), or the smaller SaCas9 that is more easily packaged into vectors (Friedland et al., 2015). Still, the feasibility of some of these systems is highly dependent on their future optimization, as well as addressing the concerns over immunogenicity and off‐target effects associated with persistent AAV‐induced expression. Self‐limiting expression systems have been proposed as one potential way to mitigate these effects (Chen et al., 2016; Li et al., 2019). Here, some of the systems with the most obvious potential as gene therapy tools for NDD + E are summarized.

#### 4.2.1 CRISPRa and CRISPRi

As discussed previously, CRISPRa/i are promising alternatives to gene supplementation. dCas9 fused to transcriptional activators or repressors are capable of adjusting endogenous gene expression and preserving normal biogenesis with a more subtle effect that is easier to titre. This could lead to superior efficacy and safety for the treatment of haploinsufficient or GoF NDD + Es. The size of these dCas9 complexes remains an issue, necessitating the use of two AAV vectors to co‐deliver the sgRNA and therefore reducing efficiency (Colasante, Lignani, et al., 2020; Colasante, Qiu, et al., 2020). Development of CRISPRa/i systems based on smaller, orthologus dCas9s, such as that produced with *Staphylococcus aureus* dCas9 (SadCas9), could be one way to overcome this issue (Gao et al., 2016). Concerns around the negative effects of long‐term dCas9 expression also exist and need further investigation before these tools are translated to clinical use.

#### 4.2.2 Base editing

CRISPR base editors are gene editing tools based on impaired Cas proteins fused to deaminases that are able to convert C‐G to T‐A and A‐T to G‐C efficiently and with exceptional precision, opening up the possibility for reliably correcting pathology at the genomic level (Gaudelli et al., 2017; Komor, Kim, Packer, Zuris, & Liu, 2016). Both classes of base editor generate minimal indels because they do not cut DNA or only generate single‐stranded nicks, making them ideal for correcting point mutations in genes with sensitive developmental roles or modelling these mutations in human cells. The size of base editor genes (6 kb) has also constrained their use in vivo because it exceeds the AAV capacity. Recently, dual AAVs have been used to deliver base editors divided into halves that are reconstituted by trans‐splicing inteins (Levy et al., 2020). Split intein base editors showed high editing efficiency in the brain when delivered systemically via IV injection of AAV‐PHP.eB vectors and were able to improve neurodegeneration in a mouse model of neurodegenerative ataxia (Levy et al., 2020). The applications of base editors have also been constrained by the narrow window of bases that they can edit relative to a PAM sequence. Newly designed base editors that recognize a wide range of non‐G PAMs open up the majority of the genome to base editing in theory, the potential increase in off‐target effects due to increasing the number of PAM sites is a concern (Miller et al., 2020; Walton et al., 2020). In fact, new tools are urgently needed to study the off‐target effects of base editors efficiently and without bias (Tang, 2020). Ultimately, before they translate to clinic, development of a construct that may be delivered in a single AAV will be required to substantially reduce the risk and cost of these approaches.

#### 4.2.3 Targeted integration

Targeted genomic integration in vivo represents an exciting means to treat monogenic NDD + E by correcting mutations while bypassing issues associated with mosaicism or dominant‐negative effects. CRISPR‐generated cleavage has been used to induce genome editing by appropriating the DSB repair process. Two repair mechanisms are known: the accurate but less efficient homology‐directed repair (HDR) and the indel prone non‐homologous end joining (NHEJ). Both pathways can be used to incorporate a DNA template, with HDR in fact requiring one that is flanked by regions of homology. While HDR is most commonly used for its accuracy, it is not very efficient in post‐mitotic cells such as neurons. An HDR approach in vivo showed that this system can be used to modify neuronal DNA, but not with high efficiency (Nishiyama, Mikuni, & Yasuda, 2017). To enable neuronal genomic integration, the NHEJ‐based technique homology‐independent targeted integration (HITI) was developed (Suzuki et al., 2016). HITI is more efficient in neurons than HDR, and was able to improve visual function by correcting a mutation in a rat model of retinal degeneration but HITI still only has a modest efficiency that several groups are attempting to improve (Gao et al., 2019; Suzuki & Izpisua Belmonte, 2018; Suzuki et al., 2019). While HITI could be used to correct large deletions underlying NDD + E, there is currently a high rate of indel introduction at the site of integration that could disrupt natural biogenesis of the target gene. The accuracy and efficiency of HITI must be improved before this approach can be translated to humans. Another technique that uses similar NHEJ integration mechanisms is homology‐independent universal genome engineering (HiUGE; Gao et al., 2019). With an efficiency in vivo and in vitro similar to HITI, HiUGE uses modular sgRNA and donor cassettes that make it ideal for high‐throughput screens labelling proteins for functional and localization analysis. HiUGE could be ideal for screening the downstream targets of NDD + E mutant transcription factors, such as *UBE3A* or *FMR1*, to identify new therapeutic targets.

Replacing a genomic locus containing a pathogenic mutation with an exogenous healthy copy early in development represents a theoretical gold standard for therapeutic genome editing in NDD + E. Prime editing is a recently developed methodology that could eventually translate to this sort of locus replacement (Anzalone et al., 2019), using an impaired Cas9 fused to a reverse transcriptase and programmed by a prime editing gRNA (pegRNA) that specifies the target and the edit template. Prime editing was shown to be efficient and precise in human cells in vitro and was able to correct the genetic causes of two diseases (Anzalone et al., 2019). Prime editing also offers a greater flexibility compared to base editing as it is less constrained by proximity to the PAM and can perform a greater range of more complex edits. Still, the efficiency of prime editing varied greatly based on the pegRNA, genomic locus and cell type, with the efficiency at two loci edited in primary neurons being low (4% and 6%). More research is needed to elucidate the cellular determinants of prime editing efficiency in order to adapt the system for higher efficiency in neurons before it can be applied to NDD + E, although it still holds exciting potential for many NDD + E diseases. For example, prime editing could in theory be used to replace the hyperexpanded CGG repeat in FMR1 to treat Fragile X syndrome or rewrite large sections of *SCN1A* to treat multiple causes of DS. A major hurdle to using prime editing in vivo is the size of prime editors and a pegRNA (around 7 kb), which far exceeds the packaging limit of AAV. It is possible that a split intein approach could overcome this as it has for base editing, but prime editing is still a very nascent technology that needs optimization.

#### 4.2.4 Editing the epigenome and RNA

Cas proteins have also been fused to a variety of epigenomic effectors, such as DNA methylases (dCa9‐Dnmt3a) or demethylases (Tet1‐dCas9) (Liu et al., 2018) to enable epigenome editing. Epigenome editing could be a powerful tool for monogenic NDD + E disorders where the epigenome is implicated, such as FXS, without introducing genomic off‐targets. Indeed, using a dCas9 fused to the Tet1 demethylase, Liu et al. were able to reverse hypermethylation of the CGG repeat in FXS iPS cells and reactivate FMRP expression (Liu et al., 2018), circumventing the risk associated with genome editing. This restoration of expression was also sufficient to rescue electrophysiological deficits, providing hope that epigenomic editing in vivo could correspond to an improvement in disease phenotype.

The Cas13 family is unique among the Cas proteins for targeting and cleaving RNA instead of DNA, allowing the CRISPR system to be applied for RNA editing (Shmakov et al., 2015). Current Cas13 applications include standard cleavage as well as the more precise base editing of C to U (Abudayyeh et al., 2019) or A to I (Cox et al., 2017). RNA editing offers potential advantages over DNA editing in correcting NDD + E pathology, with the reversibility of RNA editing being particularly appealing. In particular, RNA base editing could be promising for RTT, where the correction of a patient RTT mutation in mice by an ADAR2‐based chimeric ‘editase’ is capable of restoring *Mecp2* transcript and MeCP2 protein function (Sinnamon et al., 2020). This paper is focused on the molecular changes, and does not report on the effect of the therapy on the RTT phenotype. An additional concern is the high‐level off‐target edits, which is related to the expression of the editing tool, and remains a concern in the field.

#### 4.2.5 Inactivating CRISPR

While CRISPR/Cas is extremely precise for a genome editing tool, there are concerns around the potential deleterious effects of off‐target editing (Merkle et al., 2015; Zhang, Tee, Wang, Huang, & Yang, 2015), in particular because long‐term Cas protein expression is associated with increased off‐target editing (Merkle et al., 2015). A variety of CRISPR inhibitors called anti‐CRISPRs have been posited as an ‘off‐switch’ to limit these off‐target effects, although they are currently not developed enough to be applied for meaningful control of genome editing (Marino, Pinilla‐Redondo, Csorgo, & Bondy‐Denomy, 2020), ultimately clinical translation of CRISPR‐based tools may require some consideration of how they can be inactivated or expressed transiently.

## 5 The next steps for developing gene therapy for NDD + E

Innovative therapeutic approaches have recently been developed that aim to tackle NDD + E pathologies, alongside some that are still ongoing. The constant improvement in gene therapy tools over recent years is one of the most important reasons for this. The gene editing revolution with the discovery of CRISPR as well as the enhanced reliability and efficacy of ASOs and gene delivery have opened new ways for designing treatments. Furthermore, the increased understanding of the genetic causes of NDD + E and the possibility to build diverse models more efficiently has significantly improved the development of rational approaches.

**Figure 2:**
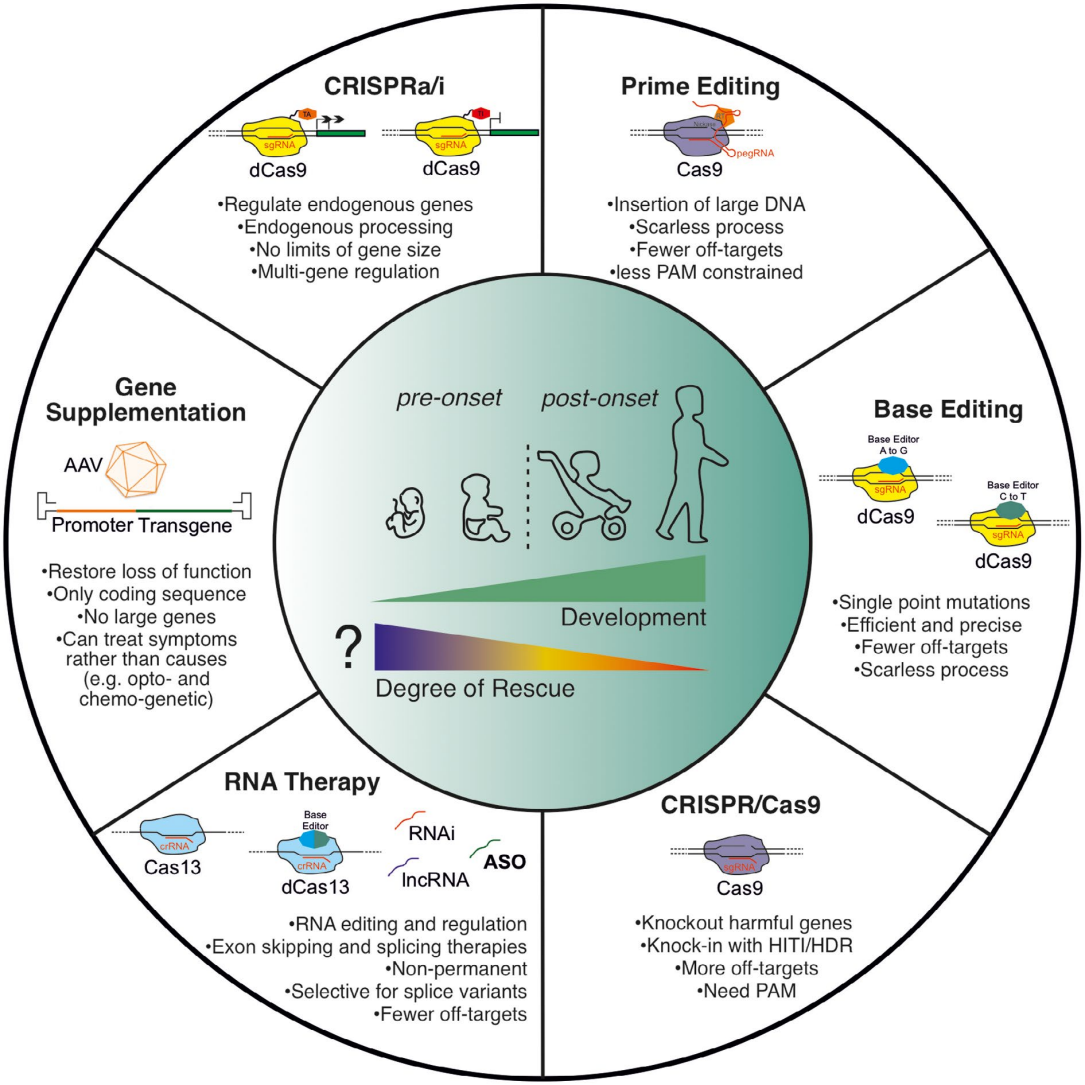
Potential gene therapy approaches for NDD + E. The onset of the disease is drawn in the early childhood but can vary among the different NDD + E

Now, we still need to better understand how NDD + E progress during development and what the best therapeutic window is for each individual disease. Rather than having a specific gene therapy tool for each single mutation or gene, we would ideally need universal tools that can cover multiple patients, and most importantly the tool should be decided based on when the intervention is feasible (Figure 2). The use of gene editing to correct a mutation at later stages of NDD + E may not result in a complete rescue of the pathology due to developmental changes that have already occurred, and it is probably preferable to intervene at or before the onset of symptoms. An early identification of the genetic causes, while not always feasible, could be a game changer for these gene therapy approaches.

## CONFLICT OF INTEREST

The authors declare no conflict of interests.
